# Relationships Between High Dietary Inflammatory Index Scores and Intestinal and Blood–Brain Barrier Integrity in the Context of Neurodegenerative Diseases

**DOI:** 10.3390/nu18132106

**Published:** 2026-06-28

**Authors:** Dariusz Szukiewicz, Juliana Almeida-de-Souza, Małgorzata Gryka-Marton, Mateusz Wątroba, Anna D. Grabowska

**Affiliations:** 1 Department of Biophysics, Physiology & Pathophysiology, Faculty of Health Sciences, Medical University of Warsaw, 02-004 Warsaw, Poland; mgrykamarton@gmail.com (M.G.-M.); mateusz.watroba@wum.edu.pl (M.W.); anna.sepulveda@wum.edu.pl (A.D.G.); 2 CIMO, LA SusTEC, Instituto Politécnico de Bragança, Campus de Santa Apolónia, 5300-253 Bragança, Portugal; julianaalmeida@ipb.pt

**Keywords:** dietary inflammatory index, intestinal barrier integrity, blood–brain barrier integrity, neurodegenerative diseases, chronic low-grade inflammation, resoleomic disorders, inflammatory markers, biomarkers of inflammation

## Abstract

The impact of diet on human health is constantly being researched. Nutrition is one of the most powerful tools for influencing gene expression, and dietary habits can promote the expression of genetic predisposition to obesity, diabetes, cardiovascular disease, cancer, and neurodegenerative diseases (NDs). The dietary inflammatory index (DII) is a numerical score that assesses the pro-or anti-inflammatory potential of a given diet. According to high DII scores, a Western diet or a standard American diet (SAD) has proinflammatory properties. By disrupting the gut microbiome, SAD creates an unfavorable environment in the intestine that is associated with a low-grade systemic inflammatory response and oxidative changes that may promote the development of NDs. An increased intestinal permeability and loss of blood–brain barrier (BBB) integrity play key roles in the pathomechanisms of diet-dependent NDs, leading to proinflammatory signaling via the gut–brain axis. The aim of this narrative review is to present in detail the current state of knowledge on the function of the gut–brain axis depending on the pro-/anti-inflammatory potential of the diet, measured by the DII, in the context of the contributions of intestinal and BBB permeability disorders to the development of NDs.

## 1. Introduction

Diet is a highly modifiable environmental factor that significantly influences an individual’s health [1]. At the level of the intestinal barrier, nutritional factors influence the diversity and functionality of the gut microbiota, in consequence directly affecting its homeostasis [2]. Nutrients and their associated metabolic processes can influence gene activity and gene expression via epigenetic mechanisms, such as DNA methylation and histone modification [3,4,5]. Gene regulation through diet significantly influences protein production and function, which translate into the control of processes crucial for cellular differentiation, development and responses to environmental stress [6]. Therefore, dietary habits can mitigate or exacerbate genetic predispositions to conditions such as obesity, diabetes, cardiovascular disease, cancer and neurodegenerative diseases (NDs) [7].

A person’s diet can provide both pro-and anti-inflammatory components, which, in the long term, can impact overall health, including the functioning of the nervous system [8,9]. A Western diet, also known as the standard American diet (SAD), typical of most industrialized Western countries (e.g., the United States Australia and some European countries) is becoming increasingly common in other parts of the world because of rising incomes. Unfortunately, SAD is associated with increased consumption of highly or ultra-processed foods (UPFs), red meat, refined grains, sugar-sweetened beverages, and fried foods but limited consumption of fiber, whole grains, fruits, and vegetables [9]. The adverse health effects of SAD, such as increased body mass index (BMI), higher triglyceride levels and higher risks of heart disease, metabolic syndrome and cancer, especially colon cancer, are well known [10]. By disrupting the gut microbiome, SAD creates an unfavorable environment in the intestine that triggers a low-grade systemic inflammatory response (manifested by increases in the blood levels of certain inflammatory markers, such as C-reactive protein (CRP), interleukins (e.g., IL-6), and tumor necrosis factor-alpha (TNF-α) and oxidative changes that may promote the development of NDs [11,12,13]. On the other hand, several healthy dietary patterns have been shown to have opposite effects, namely, reducing systemic inflammation. Most studies have focused on the Mediterranean diet, characterized by a high intake of plant-based foods, such as fruits, vegetables, beans, nuts, cereals that are minimally refined, and olive oil used as the principal source of fat; moderate consumption of animal foods, such as dairy products, eggs, fish, and poultry, and of wine accompanying the meals; and, ultimately, a low intake of red meat [14]. A recent meta-analysis revealed that high adherence to the Mediterranean diet lowers the levels of several inflammatory markers, such as IL-6, IL-1β, and CRP [15], because of the presence of significant amounts of antioxidants, vitamins, minerals, omega-3 fatty acids and polyphenols from foods [16].

The dietary inflammatory index (DII), a numerical score that assesses the pro-or anti-inflammatory potential of a given diet, was developed based on scientifically documented knowledge of the proinflammatory effects of various foods and dietary components [16]. High, positive DII values indicate a diet with proinflammatory properties, the main representative of which is SAD, while negative DII values obtained for the Mediterranean diet indicate its potent anti-inflammatory properties [16]. The DII value of a specific diet is related to the somatic and mental functions of the body, especially in connection with aging [17].

The relationship between DII values and predicting the risk of developing NDs stems from the presence of the gut–brain axis, a two-way communication system that connects the brain and the gut through metabolic, endocrine, and immune pathways [18,19,20]. In this bidirectional interaction, the gut microbiota plays a leading role on the intestinal side [21,22]. Dysbiosis or a state of microbial imbalance is correlated with the onset of NDs, such as Alzheimer’s disease (AD), Parkinson’s disease (PD), Huntington’s disease (HD) and amyotrophic lateral sclerosis disease (ALS) [23].

On the cerebral side, maintaining the integrity of the blood–brain barrier (BBB) is essential for ensuring cellular homeostasis within the central nervous system (CNS) [24]. Under conditions of increased oxidative stress, excess free radicals, including reactive oxygen species (ROS), present in the blood can damage the BBB itself, compromising its protective function and leading to increased permeability and neuroinflammation, which potentially contribute to NDs [25].

**Figure 1:**
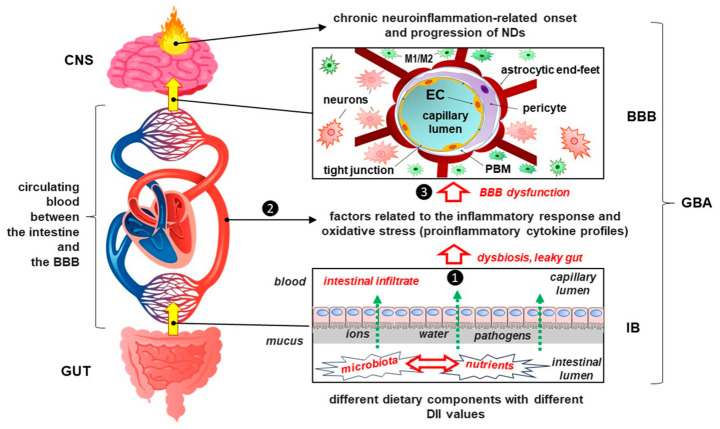
Gut–brain axis (GBA) interactions as a possible cause of chronic neuroinflammation and increased risk of neurodegenerative diseases (NDs) [7,8,18,19]. ❶ Diet-related proinflammatory factors affect the gut microbiota, which may disrupt the integrity of the intestinal barrier (IB) and lead to an increased systemic inflammatory response, reflected in ❷ increased levels of proinflammatory cytokines and oxidative stress markers in the blood. Such a proinflammatory environment at the blood–brain barrier (BBB) ❸ may degrade tight junction proteins between brain endothelial cells (ECs), allowing immune cells and other substances to uncontrollably infiltrate the central nervous system (CNS). DII—dietary inflammatory index, M1/M2—microglia classified as either proinflammatory and neurotoxic (M1) or anti-inflammatory and neuroprotective (M2), PBM—parenchymal basement membrane.

The aim of this paper is to present in detail the current state of knowledge on the function of the gut–brain axis depending on the pro-/anti-inflammatory potential of the diet, as measured by the DII, in the context of the contributions of intestinal and BBB permeability disorders to the pathomechanisms of NDs. Unlike other reviews presenting an integrated approach to the involvement of the intestinal barrier and the BBB in the development of NDs, the authors draw attention to the DII as a practical tool for assessing the inflammatory potential of the diet. Considering the broad cross-sectional nature of this work and the lack of literature that would provide a synthesis of all the issues discussed, we considered the narrative review form to be optimal. The issues raised in the review are illustrated in Figure 1.

## 2. Methodology Used for the Literature Search

A comprehensive analysis of the effects of dietary inflammatory potential, considering the gut microbiome and intestinal barrier permeability, on the levels of proinflammatory factors and oxidative stress markers in the blood was performed in the context of the development of BBB integrity disorders and chronic neuroinflammation, which promote the development of NDs. While trying to maintain maximum criticism and objectivity, a synthesis of relevant research and generalization of the collected data was performed. This approach established a coherent framework for presenting the findings, supporting a qualitative analysis of the topic and facilitating the achievement of the predefined objectives. The analysis focused primarily on the link between the physiological/pathophysiological mechanism of the influence of dietary factors with different inflammatory potentials on the risk of developing NDs. In this respect, the components of the gut–brain axis, such as the intestinal microbiome, the functional state of the intestinal barrier, the levels of inflammatory and oxidative stress markers in the blood, the functional state of the BBB and the associated development of neuroinflammation in the brain tissue, were analyzed. This approach enabled the identification of pathways and factors involved in the disruption of both intestinal barrier function and BBB function and the development of neuroinflammation.

Highly cited, peer-reviewed publications in journals from the Master Journal List (MJL) in the fields of anatomy, physiology/pathophysiology, nutrition and dietetics, gastroenterology, immunology, neurology/neuropathology and bacteriology were selected. An important selection criterion for review articles was their topicality (date of publication). The electronic databases PubMed, Scopus, Web of Science (WoS) and Google Scholar were reviewed. Priority was given to studies providing observational or experimental evidence. The core of the literature search consisted of publications within the time frame of 2015–2026, and in the case of review articles with the same topic defined by keywords, publications from journals positioned in Q1 were preferred. During manuscript preparation, the citation list was then supplemented with key milestone articles that captured the phenomena and the biologically active compounds properties, cited regardless of publication date.

The general scope of the literature included the following:

- The papers on the structure and function of the intestinal barrier and the importance of the intestinal bacterial flora (gut microbiome), identified using the following leading keywords/phrases: “intestinal mucosal barrier”, “gut microbiota and intestinal permeability”, “gut dysbiosis and inflammatory mediators”, “gut dysbiosis and oxidative stress” and “gut microbiota in health and disease”;
- Research data on the DII and its association with the risk of loss of intestinal barrier integrity, increased concentrations of markers of the inflammatory response and oxidative stress in the blood, and BBB dysfunction, obtained using the following leading keywords/phrases: “dietary inflammatory index”, “dietary inflammatory index and inflammatory response”, “intestinal barrier integrity and inflammatory markers in blood”, “proinflammatory diet and blood cytokine patterns”, “leaky gut”, “gut–brain axis”, “inflammatory response and blood–brain barrier function” and “blood–brain barrier and neuroinflammation”;
- Sources exploring the relationship between chronic systemic inflammation and neuroinflammation in the CNS and an increased risk of NDs were chosen from research combining theoretical models with experimental findings. The relevant leading keywords/phrases included “neuroinflammation and neurodegenerative diseases”, “inflammatory response and neurodegenerative diseases”, “immune activation and central nervous system” and “inflammatory background of neurodegenerative diseases”.

A synthesis of the collected data was conducted to elucidate the mechanisms by which proinflammatory factors, including nutrients, interact with the gut–brain axis to trigger the pathomechanism of NDs. The findings were then summarized and generalized to highlight and present potential strategies to mitigate neuroinflammation induced by the BBB penetration of diet-induced proinflammatory factors.

**Figure 2:**
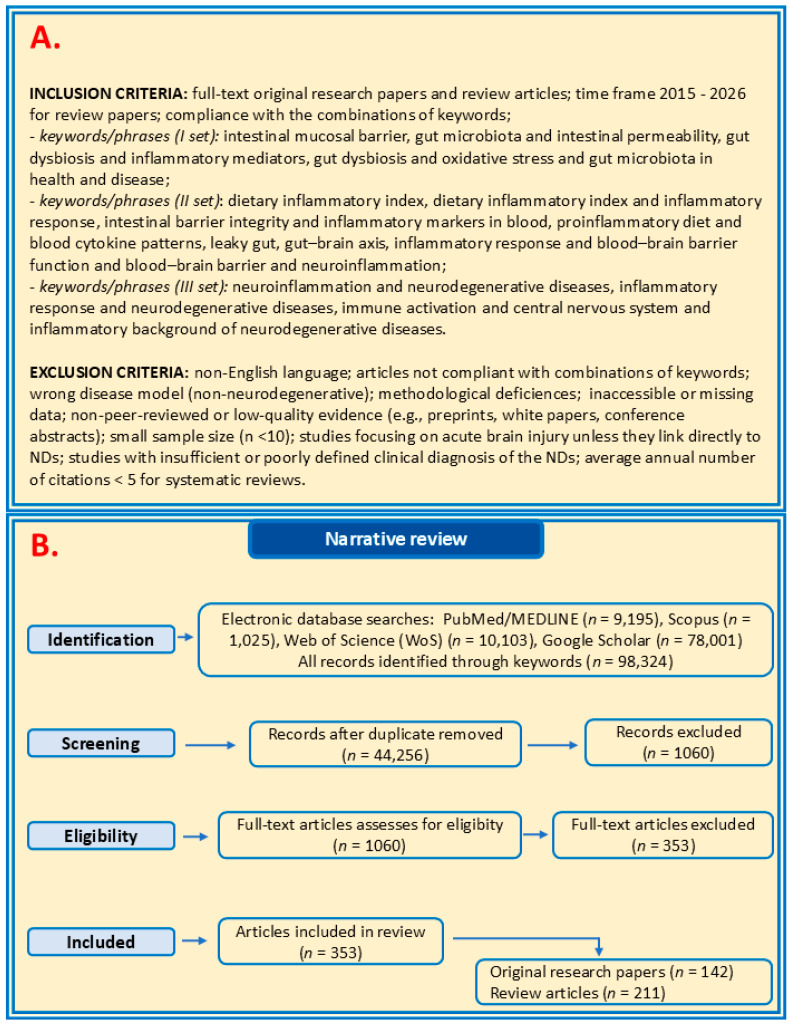
Literature search methodology: (**A**)—article inclusion/exclusion criteria; (**B**)—flowchart diagram of the literature review procedure.

The literature retrieval methodology is summarized in Figure 2.

## 3. Influence of the Diet on Inflammation in the Body

### 3.1. Resoleomic Disorders and Chronic Low-Grade Inflammatory Response

A diet rich in omega-6 fatty acids (primarily linoleic acid) is, in addition to long-term psycho-emotional stress and the chronic overuse of anti-inflammatory medications, the main cause of the breakdown of the physiological mechanisms controlling the course of the inflammatory response. These include the process of inflammation resolution, referred to as “resoleomics” that allows the restoration of homeostatic balance after injury, inflammation and infection [26,27]. This active, self-limiting and highly orchestrated process involves immune cells producing specialized proresolving mediators (SPMs) such as resolvins, protectins, and maresins from omega-3 fatty acids [eicosapentaenoic acid (EPA) and docosahexaenoic acid (DHA)] to stop neutrophil infiltration by inhibiting chemotaxis and promote healing [28,29]. In addition, EPA and DHA reduce adhesion molecule expression and leucocyte–endothelial adhesive interactions and limit the production of proinflammatory cytokines and eicosanoid lipid mediators such as prostaglandins (PGs) and leukotrienes (LTs) from the omega-6 fatty acid arachidonic acid (ARA) [30]. It is worth noting that omega-6 polyunsaturated fatty acids (PUFAs), perceived in the resoleomic model as a proinflammatory factor in the diet, are assigned a negative overall inflammatory score (−0.159) in the DII (see Table 1 in Section 3.3; Dietary Inflammatory index (DII)), indicating an anti-inflammatory effect. This apparent discrepancy stems from the fact that PUFAs, primarily linoleic acid (LA), are essential dietary precursors that the body converts into ARA. Only after this transformation, although essential for health, in high concentrations ARA usually causes overproduction of proinflammatory mediators (e.g., PGs and LTs) [30].

Under physiological conditions, resoleomics using the innate immune system (IIS) and regulated by the sympathetic nervous system (SNS) and the hypothalamus–pituitary–adrenal (HPA) axis should be able to elucidate inflammatory responses. However, a proinflammatory diet, combined with physical inactivity and subsequent obesity, including visceral obesity, the resoleomics becomes ineffective, leading to chronic low-grade inflammation (CLGI) associated with chronic inflammatory diseases [10,31,32]. CLGI inherently constitutes to inflammaging, defined as a chronic, progressive, and systemic low-grade, sterile (non-infectious) and often silent inflammatory state that exacerbates with age [32,33].

### 3.2. Markers of the Low-Grade Inflammatory Response

Unlike acute inflammation, chronic inflammation is a long-term, low-grade (subclinical) immune response and systemic process that lasts for months or years [34]. Both acute and chronic inflammation involve common mediators, such as tumor necrosis factor-alpha (TNF-α), interleukin-1 (IL-1), and interleukin-6 (IL-6), and even—despite being a classic acute-phase marker—C-reactive protein (CRP) [35,36]. Although chronic inflammation can often be a consequence of acute inflammation, CLGI can precede the acute process or can be completely unrelated to it, as it occurs in noncommunicable autoimmune chronic inflammatory diseases such as rheumatoid arthritis, diabetes, cancer, autoimmune disorders, obesity, and inflammatory bowel disease (IBD) [37,38].

The search for optimal CLGI markers has become the focus of numerous studies [39,40,41]. A good biomarker of chronic inflammation should be biologically stable with a relatively long half-life, accurate by demonstrating high sensitivity to low-grade inflammation, reliable, provide valuable diagnostic/prognostic clinical utility and minimally affected by short-term factors, distinguishing it from acute markers. In clinical practice, a good marker should be measurable using minimally invasive procedures, such as routine blood, urine or saliva sampling [41,42,43]. Currently, high-sensitivity C-reactive protein (hs-CRP) and the erythrocyte sedimentation rate (ESR) are clinical mainstays for detecting systemic inflammation, even though they can be swayed by acute issues.

Finally, despite defining CLGI as a persistent elevation of systemic inflammatory biomarkers in the absence of acute infection or injury, there are no universal diagnostic criteria for this condition [40]. Therefore, different studies use different biomarker combinations, thresholds, and clinical frameworks. Comparing the results of such studies and drawing clear conclusions may be difficult [40,41].

#### 3.2.1. Key Blood Biomarkers of CLGI

The key and most commonly used markers of CLGI include CRP, IL-6, TNF-α, glycoprotein acetylation (GlycA), white blood cell (WBC) count, ESR and plasma viscosity (PV) [44], the latter being usually analyzed in conjunction with ESR and CRP level [45]. In the course of CLGIs, the observed increases in the levels of the above markers are of a fixed nature, and the assessed/measured values are typically lower compared to the high spikes observed in acute infections [46].

It should be noted that the ranges of values and/or threshold values given based on literature data for the blood biomarkers of CLGI characterized below are only indicative. Due to the significant heterogeneity of research methods, cut-off points, population characteristics and clinical interpretation in different studies, they cannot constitute a universal reference point in medical practice without prior clinical standardization [40,41]. The reproducibility of the results of determining blood biomarkers of CLGI in the same patient may be influenced to a varying extent by factors such as the level of physical activity, metabolic adaptation, tissue repair, and activation of the immune system or immunosuppression superimposed on CLGI [35,37,41]. Furthermore, both IL-6 and TNF-alpha are unique, highly pleiotropic signaling proteins. While they are both well-known triggers of the body’s immune response to infection and injury, they also possess distinct anti-inflammatory mechanisms that help prevent immune overreaction [42].

1. **CRP**

CRP belongs to the family of small (short) pentraxins, highly conserved components of the humoral innate immune system [47]. CRP is produced primarily and almost exclusively in the liver by hepatocytes as part of the acute-phase response to inflammation, infection or tissue damage, upon release of proinflammatory cytokines, especially IL-6, along with IL-1β and TNF-α [48]. 

After signal peptide being cleaved, CRP is found in the circulatory system, mainly as an annular pentamer consisting of nonglycosylated and noncovalently associated 23 kDa protomers [49]. CRP-induced processes encompass a wide spectrum of overlapping phenomena in the circulatory system, including the activation of the complement cascade with the development of inflammation and a prothrombotic state, endothelial dysfunction and oxidative stress, vascular wall damage and plaque remodeling with opsonization of oxidized low-density lipoproteins (LDL) resulting in the formation of foam cells [50,51]. Increased levels of CRP are also associated with chronic pain independent of biopsychosocial factors [50].

In CLGI diagnostics, the measurement of the high-sensitivity CRP (hs-CRP) level is used to determine the CRP concentration, which facilitates the detection of very small amounts of this protein [52]. hs-CRP is a powerful indicator of chronic inflammation and the cardiovascular risk [52]. The optimal hs-CRP concentration is set at less than 1 mg/L. In individuals with metabolic inflammation (metainflammation), hs-CRP levels are chronically slightly elevated, typically ranging between 1.0 and 3.0 mg/L, higher in patients with type 2 diabetes and metabohlic syndrome and often reach even >10 mg/L in patients with metabolic dysfunction-associated steatotic liver disease (MASLD) [52].

2. **IL-6**

IL-6 plays a central role in driving persistent inflammatory conditions. The human IL6 gene encodes a 21–26 kDa glycoprotein produced by macrophages, T and B cells, fibroblasts, and endothelial cells (ECs) in response to infections, injuries, or presence of other cytokines (e.g., IL-1β and TNF-α) [53]. 

Classical signaling occurs through membrane-bound interleukin-6 receptor (IL-6R) on immune cells and hepatocytes, whereas trans-signaling, which often drives chronic inflammation, involves the binding of soluble IL-6R (sIL-6R) to act on cells lacking IL-6R [54,55].

In individuals with CLGI, serum IL-6 levels are slightly increased and typically range from 2.5 pg/mL to 5 pg/mL, compared to values below 1–2 pg/mL in healthy individuals [56].

3. **TNF-α**

Human TNF-α (former name: cachectin, TNFSF1A) is a highly polymorphic cytokine that occurs in two distinct biologically active forms: membrane-bound (mTNF-α) and soluble (sTNF-α) [57]. sTNF-α binds primarily to tumor necrosis factor receptor 1 (TNFR1) and plays an important role in the inflammatory immune response, whereas mTNF-α interacts primarily with tumor necrosis factor receptor 2 (TNFR2) and promotes cellular proliferation and survival and other less known effects [57]. 

TNF-α is one of the most important and ubiquitous cytokines in the human body produced primarily by activated macrophages and monocytes during the acute phase of inflammation, infection, or injury [58]. 

Blood TNF-α levels are highly stable over short periods but decrease substantially over longer intervals. Therefore, single measurements adequately represent an individual’s TNF-alpha status for up to 4–6 months, but repeated measurements are necessary for accurate characterization beyond 2–2.5 years [59].

Without exogenous stimuli, TNF-α concentrations in human serum are almost always below or at the detection limit of most assays (typically approximately 3 pg/mL) and range from 0.5 pg/mL to 5.0 pg/mL, with the levels in many healthy individuals falling below 2.8 pg/mL or approximately 1–2 pg/mL [60]. TNF-α levels exceeding 15.0 pg/mL are usually considered elevated and may correspond to CLGI, whereas those exceeding 40 pg/mL indicate increased inflammatory activity [61,62,63].

4. **GlycA**

In recent years, a new method has emerged in which nuclear magnetic resonance (NMR) is used to quantify glycoprotein acetylation (GlycA), whose usefulness in assessing the intensity of inflammation is based on the analysis of the N-acetyl methyl groups of specific acute-phase proteins [64]. While hsCRP is a commonly used single marker of inflammation, GlycA is a composite biomarker that reflects the glycosylation states of several of the most abundant acute-phase proteins, including α1-acid glycoprotein, haptoglobin, α1-antitrypsin, α1-antichymotrypsin, and transferrin [64]. Compared with “traditional” markers, GlycA assay provides a more stable, comprehensive and precise measurement of a CLGI, is often linked to neutrophil activity, associated with gut microbiome diversity, and is sometimes superior to hsCRP [65]. The advantage of GlycA over hsCRP is lower intraindividual variability (greater stability over time) [65]. 

Serum GlycA concentrations predictive of CLGI are generally assumed to exceed 387–400 μmol/L (highest quartile) in humans, with the mean in the general population being approximately 352 ± 62 μmol/L [66,67,68]. The level of GlycA, as a biomarker, is highly stable over time and remains consistent within individuals for more than a decade [68].

5. **WBC count**

An increase in the production of leukocytes (leukocytosis) occurs in the bone marrow mainly in response to infection (mostly bacterial) or trauma [69]. Leukocytosis in CLGI is primarily triggered and then maintained by the sustained release of inflammatory mediators and growth factors (primarily colony-stimulating factors (CSFs)) that stimulate the bone marrow [32,70]. 

CLGI typically presents with mild, often subclinical, elevated WBC counts, frequently remaining within the upper limit of the normal range (e.g., 8500–11,000 cells/μL). This “subclinical” or relative leukocytosis is characterized by an increase in the percentage of a specific white blood cell type (usually neutrophils or lymphocytes) that may serve as a marker for metabolic, cardiovascular, and age-related, stress-related, or lifestyle-related CLGI diseases [71]. The limitations of the WBC count in the diagnosis of CLGI, resulting primarily from insufficient sensitivity, mean that other markers, e.g., hsCRP, are preferred [72].

Combining the WBC count with the neutrophil-to-lymphocyte ratio (NLR) and platelet-to-lymphocyte ratio (PLR) significantly increases the diagnostic value of low-cost blood cell tests [73]. The pathological condition corresponding to CLGI is diagnosed with values greater than 3, whereas in healthy adults, the normal NLR is typically between 1 and 2. PLR is a marker reflecting systemic inflammation and thrombotic activity, with typical normal ranges of 90–180 and elevated values >180 or >200 indicating increased inflammatory activity. A lower PLR, which is often defined as below 150, typically suggests the absence of significant inflammation corresponding to CLGI [74].

6. **ESR**

The ESR test (also known as the Biernacki reaction) measures the rate at which erythrocytes (red blood cells (RBCs)) in whole blood samples containing anticoagulant sediment to the bottom of the tube (the Westergren tube) over an hour under the influence of gravity [75]. This rate of RBC sedimentation corresponds to the distance expressed in millimeters and is directly proportional to the concentration of proteins in the blood [75]. Because both acute and chronic inflammatory states trigger the liver to increase the production of specific proteins known as acute-phase reactants, leading to elevated protein levels in the blood, ESR serves as a routine hematology test used to detect and monitor increased inflammatory activity in response to conditions such as autoimmune disorders, infections, or tumors [76].

Although not specific to a single disease, the ESR supports the evaluation of inflammation when interpreted alongside other diagnostic tests. ESR values tend to naturally increase slightly with age, and females often have higher baseline values. In CLGI, an elevated ESR is typically in the range of 20–50 mm/h or slightly above age-adjusted norms and serves as a nonspecific indicator of underlying systemic inflammatory conditions [77].

7. **PV**

Increases in the levels of acute-phase proteins (e.g., CRP, serum amyloid A, immunoglobulins, and fibrinogen) in the blood contributes to an increase in PV [45]. PV is therefore an effective, sensitive laboratory marker that reflects both acute and chronic inflammation and is generally considered superior to the ESR because it is not affected by age, sex or anemia. The standard unit for measuring the PV is millipascal seconds (mPa ∙ s), which is equivalent to the centipoise (cP) unit (1 mPa ∙ s = 1 cP) [78].

The normal PV range is 1.50–1.72 mPa⋅s at 25 °C or approximately 1.10–1.30 mPa⋅s at 37 °C. The PV values indicative of CLGI are generally in the range of 1.72–1.85 mPa⋅s or 1.30–1.70 mPa⋅s (at 25 °C or 37 °C, respectively) or are slightly higher much less frequently. PV is used to detect and monitor disease activity in patients with autoimmune, cardiovascular, and metabolic conditions [78].

#### 3.2.2. Additional CLGI-Related Indicators and Clinical Context

Experimental data show the simultaneous existence of CLGI and oxidative stress in many chronic diseases. There is a bidirectional correlation between oxidative stress markers and CLGI. As the inflammatory process can induce oxidative stress, the oxidative stress can also induce inflammation through activation of multiple pathways [79].

1. Oxidative stress markers

Oxidative stress markers are indicators of an imbalance between the production of reactive oxygen species (ROS) and the body’s antioxidant defense systems [79]. Typically, the most commonly used markers of oxidative stress are classified based on the type of molecules/structures they damage (e.g., cell membranes, genetic material, proteins) or include measurements of the enzyme myeloperoxidase (MPO) and reactive oxygen metabolites (ROMs), which allow to assess the overall capacity of the antioxidant defense system [79].

2. Metabolic factors and clinical correlates

Chronic inflammation is closely linked to specific metabolic disorders and is reflected in the term “metainflammation” [80]. In metabolic syndrome, three or more factors coexist with immune system dysfunction, such as hypertension, high fasting glucose levels, dyslipidemia [high triglyceride levels or low high-density lipoprotein (HDL) levels], a larger waist circumference (abdominal obesity) and a BMI of 30.0 kg/m^2^ or higher [81]. CLGI-related metabolic factors in the blood include insulin resistance, dyslipidemia, dysadipokinemia or adipose tissue dysfunction, procoagulants, elevated lipopolysaccharide (LPS), sustained increases in cortisol levels and elevated levels of liver enzymes and ferritin [81,82,83,84,85].

The vicious cycle of the pathomechanisms of CLGI also include specific nutrient deficiencies that hinder the body’s ability to resolve inflammation [86]. These include vitamin deficiencies (e.g., B-complex vitamins B1, B5, B6, and B12 as well as vitamin D, C and E deficiencies) [87,88,89], mineral deficiencies (iron, magnesium, selenium and zinc deficiencies) [90] and fatty acid imbalances caused by high intake of proinflammatory omega-6 fatty acids in combination with low levels of anti-inflammatory omega-3 fatty acids [91].

### 3.3. Dietary Inflammatory Index (DII) as an Indicator of Pro-/Anti-Inflammatory Effect of Diet

An evidence-based, literature-derived DII elaborated for the first time in 2009 by researchers at the University of South Carolina has become a valuable tool for classifying an individual’s diet within the theoretical range from pro-inflammatory (scores positive with a maximum of +7.98), through a score of “zero” considered neutral, to anti-inflammatory (negative scores with a minimum of −8.87) [92,93]. Based on 927 peer-reviewed articles, the effects of a given diet on the production of six markers of inflammation (CRP, IL-1β, IL-4, IL-6, IL-10, and TNF-α) have been established [70,92]. A significantly improved and widely used revised calculation of the DII was released in 2014 [93,94], based on a review of more than 1900 scientific articles. The inflammatory potential of an individual’s diet is calculated (summed) using 45 specific food parameters that encompass macronutrients, micronutrients, and whole food components (such as herbs and spices) [94]. A total of 9 proinflammatory and 36 anti-inflammatory diet components were identified (Table 1).

**Table 1:** Overall inflammatory effect scores (IES) for the proinflammatory (positive values in the red column) and anti-inflammatory (negative values in the green column) 45 dietary components used to calculate the dietary inflammatory index (DII) [94]. * RAE—retinol activity equivalent.

Food Parameter [Unit of Measurement]	Overall IES	Food Parameter [Unit of Measurement]	Overall IES
**PROINFLAMMATORY COMPONENTS (PC)** 1. Total Energy [kcal] 2. Carbohydrates [g] 3. Protein [g] 4. Total Fat [g] 5. Saturated Fatty Acids (SFAs) [g] 6. Trans Fats [g] 7. Cholesterol [mg] 8. Iron (Fe) [mg] 9. Vitamin B_12_ (cobalamin) [μg]	**PC** **0.180** **0.097** **0.021** **0.298** **0.429** **0.432** **0.110** **0.032** **0.106**	**ANTIINFLAMMATORY COMPONENTS (AC)** **Macronutrients and fibers:** 10. Dietary fiber (g) 11. Monounsaturated Fatty Acids (MUFAs) [g] 12. Polyunsaturated Fatty Acids (PUFAs) [g] 13. Omega-3 Fatty Acids (n-3 FAs) [g] 14. Omega-6 Fatty Acids (n-6 FAs) [g] 15. Alcohol [g] **Vitamins (excluding vitamin B_12_):** 16. β-carotene [μg] 17. Folic acid (vitamin B9) [μg] 18. Niacin (vitamin B3, vitamin PP) [mg] 19. Vitamin A (retinol) [μg of RAE *] 20. Vitamin B1 (Thiamin) [mg] 21. Vitamin B2 (Riboflavin) [mg] 22. Vitamin B6 (Pyridoxine) [mg] 23. Vitamin C (L-ascorbic acid) [mg] 24. Vitamin D (calciferol) [μg] 25. Vitamin E (α-tocopherol) [mg] **Minerals (excluding iron):** 26. Magnesium (Mg) [mg] 27. Selenium (Se) [μg] 28. Zinc (Zn) [mg] **Flavonoids:** 29. Anthocyanidins [mg] 30. Flavan-3-ols [mg] 31. Flavanones [mg] 32. Flavones [mg] 33. Flavonols [mg] 34. Isoflavones [mg] **Spices, herbs and other items:** 35. Garlic [g] 36. Ginger [g] 37. Onion [g] 38. Saffron [g] 39. Turmeric [mg] 40. Pepper [g] 41. Thyme/Oregano [mg] 42. Rosemary [mg] 43. Eugenol (Eugenic acid) [mg] 44. Caffeine [g] 45. Green/Black tea [g]	**AC** **−0.663** **−0.009** **−0.337** **−0.436** **−0.159** **−0.278** **−0.584** **−0.198** **−0.246** **−0.401** **−0.098** **−0.068** **−0.365** **−0.424** **−0.446** **−0.419** **−0.484** **−0.191** **−0.313** **−0.131** **−0.415** **−0.250** **−0.616** **−0.467** **−0.593** **−0.412** **−0.453** **−0.301** **−0.140** **−0.785** **−0.131** **−0.102** **−0.013** **−0.140** **−0.110** **−0.536**

In clinical practice and research studies such as the National Health and Nutrition Examination Survey (NHANES), a shortened list of food parameters (e.g., 27–29 parameters) is often used because data on the intake of all spices and specific flavonoids are difficult to collect in standard food frequency questionnaires (FFQs) [95,96,97].

The appropriate period for assessing/calculating the DII is considered one that allows the habitual diet to be captured, with four or more 24 h dietary recalls or an FFQ covering the past 3–6 months or up to a year [98]. A review of studies shows that at least 3 to 4 days of food records (including weekends) are often used to estimate usual intake, providing a reliable DII score comparable to that of longer-term questionnaires. Regardless of the period (e.g., daily, 3-day, monthly, half-yearly or annually), the DII uses standardized, energy-adjusted dietary data to evaluate the inflammatory potential of the diet [99,100].

While the DII in its “classic” version takes into account six markers of inflammation (CRP, IL-1β, IL-4, IL-6, IL-10, and TNF-α), researchers have attempted to supplement it by assessing the serum concentration of GlycA and the WBC count (also considering NLR and PLR), as well as the ESR and PV [101,102,103,104,105].

### 3.4. DII Limitations

Reasonable use of the DII for assessing the inflammatory potential of a person’s diet requires acknowledgement of its limitations, resulting from the way of DII development, data collection methods and the complex nature of nutrition science [106]. Factors that reduce the reliability of the DII include the following:

- **Limitations of the Original DII Algorithm**, which often requires arbitrary scaling to work (e.g., dividing or multiplying nutrients by 10 or 100), causing distortions in the data [94];
- **Dependency on self-reported data**, making FFQs or 24 h dietary recalls susceptible to recall errors and reporting integrity [97];
- **Missing/limited dietary parameters** in some studies (e.g., 25–28 of the 45 possible), which means that the calculated DII may not fully reflect the complexity of an individual’s dietary intake (e.g., omitted specific spices, herbs, and other specialized foods that are potent anti-inflammatory modulators) [97];
- **Limited representation of cultural diets**, due to the fact that although the DII is derived from a global database, it strongly reflects Western dietary patterns and may not fully capture the anti-inflammatory power of spices, herbs, and unique foods consumed in Asian or other non-Western cultures, leading to potential inaccuracies in those populations [97,102,105];
- **Inability to reflect lifestyle factors,** where focusing solely on diet causes overlooking other key lifestyle factors that contribute heavily to chronic inflammation, such as physical activity, smoking and sleep patterns [100];
- **Focusing on nutrients rather than whole foods**, which ignores the fact that the health effects of a diet are often driven by the synergy of whole foods rather than the sum of individual nutrients [93];
- Limitations of applying population-derived inflammatory scores to individual patients [94].

The above limitations of DII may contribute to the differences between observational associations and causal inference.

DII score calculations are complex and require detailed dietary data. Serious concerns about the impact of an arbitrary way of assessing nutrient intake on DII scores have been significantly reduced in the latest, improved version of the DII [92,93,94]. However, the DII is often highly correlated with other dietary quality scores (e.g., the Healthy Eating Index), making it difficult to isolate whether it is the specific inflammatory aspect or just general healthy eating that is reducing disease risk [93,99].

Despite these limitations, the DII remains a useful tool for highlighting the impact of inflammatory diets on chronic diseases like type 2 diabetes, cancer, depression, as well as on a group of chronic, progressive disorders characterized by the gradual loss of structure and function of neurons, which include NDs [93,96,97]. Nevertheless, the scientific community suggests that future studies should aim to integrate the DII with other lifestyle factors and rely on more comprehensive dietary data to strengthen its findings [93,94].

A significant improvement in the reliability of the DII was achieved by introducing The Energy-Adjusted Dietary Inflammatory Index (E-DII), designed to evaluate the inflammatory potential of a diet regardless of the total amount of food consumed [106,107]. The most common way of calculating E-DII is by using the energy density method, where all dietary parameters (foods, nutrients) are converted to a standard unit, usually amount per 1000 kcal. To calculate E-DII, food parameters are often converted to a centered percentile score to minimize right-skewing, then multiplied by the inflammatory effect score, and summed [107,108]. In the residual method of E-DII calculation, energy-adjusted intake is determined by a regression model. A regression model is built with the absolute nutrient intake as the dependent variable and total energy intake as the independent variable. The residual (difference between observed and expected intake) is used as the adjusted value. This approach, in the regression model, effectively removes variance in nutrient intake that is caused by total energy intake [108,109]. Another, the partition model of E-DII calculation, simply adjusts for “remaining energy” (total caloric intake excluding the specific nutrient of interest) [109].

## 4. Impact of Long-Term Proinflammatory Dietary Patterns on Intestinal Barrier Integrity

Specific eating habits that have been established and repeated over a sufficiently long and individually varied period may influence the permeability of the intestinal barrier [110]. In the context of CLGI development, the decisive factors are disorders leading to the loss of selectivity of this barrier, which modulates the absorption of nutrients, water, and electrolytes and increases the risk of undesirable passage of harmful factors such as pathogens (bacteria, viruses, fungi, and prions), toxins, and food antigens from the gut lumen into the bloodstream [111]. Thus, the essential function of the intestinal barrier as the body’s primary defense in maintaining immune homeostasis and preventing systemic inflammation is compromised [110]. DII values corresponding to a proinflammatory diet (e.g., SAD) correlate with the disruption of the intestinal barrier or increased intestinal permeability (colloquially referred to as “leaky gut”), causing systemic inflammation and increased risks of IBD, obesity and diabetes [111].

### 4.1. Mechanisms Regulating Intestinal Barrier Integrity

The most important components of the intestinal barrier include the intestinal epithelium (enterocytes interconnected by tight junctions), the mucous membrane, the immune system and the gut microbiota [112].

#### 4.1.1. Enterocytes and Tight Junctions (TJs)

A complex system of connections between a single layer of epithelial cells (enterocytes) and junctional complexes creates the physical/mechanical barrier between epithelial and endothelial cells on which paracellular permeability depends [113]. TJs, also known as zonula occludens, are belt-like intercellular connecting structures located at the most apical part of adjacent epithelial cells [113]. TJs consist of specialized proteins that form a selectively permeable network composed of continuous threads of claudins (acting as “pores” or “seals”), occludins, junctional adhesion molecules (JAMs) and tricellulin. The above transmembrane junctional proteins connect to the actin cytoskeleton via cytoplasmic scaffolding proteins or intracellular adapter proteins (ZO-1, -2, and -3), which provide structural stability [113]. Tricellulin, as its name suggests, constitutes a crucial molecular link at the vertices of epithelial cells, where three cells (tricellular TJs) contact each other and ensure that the intercellular gap is closed at the corners of cells, preventing the leakage of large molecules [114].

Essentially, the regulation of intestinal TJ permeability is mediated by several key growth factors, the actions of which cause posttranslational modifications (phosphorylation) of TJ proteins by kinases [e.g., protein kinase C (PKC)] or signaling pathways [e.g., mitogen-activated protein kinase/extracellular signal-regulated kinase (MAPK/ERK) or myosin light chain kinase (MLCK)], leading to the opening or sealing of the barrier [115,116]. Transforming growth factor-β (TGF-β) maintains TJ barrier integrity by increasing basal transepithelial electrical resistance (TEER) and reducing the permeability of pathogens (e.g., *Escherichia coli*) [117,118]. Epidermal growth factor (EGF) also acts as a sealant, promoting and maintaining repair/regeneration of the intestinal epithelial barrier [119]. Conversely, inflammatory cytokines such as tumor necrosis factor-α (TNF-α), interferon-γ (IFN-γ) and interleukins-1β (IL-1β), -4 (IL-4) and -13 (IL13) typically increase the permeability of the barrier by downregulating proteins such as ZO-1 or upregulating claudin-2 [120,121].

Notably, adherens junctions (AJs), which are primarily composed of the transmembrane proteins E-cadherin and nectins, and desmosomes, which form crucial cadherin-based intercellular complexes that strongly connect adjacent cells and maintain tissue integrity and structure to influence cell shape by binding to the actin cytoskeleton, are present below TJs [122,123]. Furthermore, gap junctions (GJs) play important roles in communication between enterocytes [124]. Functionally, GJs in animal cells correspond to plasmodesmata in plant cells: they are specialized channels between adjacent intestinal cells that allow the direct transfer of small molecules (<1 kDa), facilitating intercellular communication, ion homeostasis and volume control [125]. In vertebrates, GJs are primarily composed of the membrane protein connexin-43 (Cx43) that forms hexameric, cylinder-like structures known as connexons (also called hemichannels). GJs are crucial for coordinating enterocyte migration along the crypt–villous axis and promoting intestinal mucosal healing [126].

#### 4.1.2. Intestinal Mucosal Barrier

The gastrointestinal epithelium is covered with mucus, the layer of which varies significantly depending on the location within the gastrointestinal tract and the method of measurement, generally ranging on average from 100–500 μm in the small intestine to 100–850 μm in the human colon [127]. The mucus produced by goblet cells mainly contains mucin 2 (MUC2), the main mucin that forms a gel whose volume increases 100–1000-fold after release [128]. This layer prevents direct contact between bacteria and intestinal cells [129]. Moreover, Paneth cells located in small intestinal crypts secrete a variety of antimicrobial peptides (AMPs) and proteins to preserve stem cells and regulate the gut microbiota. These peptides, along with enzymes such as lysozyme and secretory phospholipase A2 (sPLA2), bind to mucin glycoproteins (mainly MUC2) to protect the epithelium [130]. This chemical/biochemical mucosal barrier is also supported by secretory IgA antibodies (sIgA), which neutralize pathogens and toxins and simultaneously modulate interactions with commensal bacteria from the microbiome [131].

#### 4.1.3. Intestinal Immune System

The intestinal immune system acts as a crucial, dynamic immunological barrier, housing 70–80% of the immune cells in the body to manage interactions between the microbiome and host [132]. The lamina propria contains large numbers of immune cells, including the most efficient antigen-presenting cells (APCs), dendritic cells (DCs), as well as macrophages (Mφs) and lymphocytes, to maintain homeostasis and combat invading pathogens [132].

Toll-like receptors (TLRs) are located at multiple sites within the intestinal barrier, including the surface of enterocytes, intracellular compartments of enterocytes, and on immune cells within the underlying lamina propria [133]. Their location is specialized to distinguish between beneficial commensal bacteria and invasive pathogens, thereby maintaining homeostasis or initiating an inflammatory response, respectively. 

#### 4.1.4. The Gut Microbiota and Its Metabolites

Bacteria are the most numerous and well-studied group of microorganisms in the human intestine, comprising more than 1000 different species, with major phyla including *Firmicutes*, *Bacteroidetes*, *Proteobacteria*, and *Actinobacteria* [134]. *Firmicutes* and *Bacteroidetes* dominate the gut microbiota in humans, collectively constituting more than 90% of the total microbial community. They play crucial roles in metabolism, immune system maturation, and pathogen protection [135].

Due to the presence of various enzymes, the gut microbiota can participate in the metabolism and absorption of many nutrients that would otherwise be nonabsorbable [e.g., the breakdown of complex dietary polysaccharides, fermentation of ingestible dietary fiber into short-chain fatty acids (SCFAs) [136], and the biosynthesis of amino acids, including essential amino acids (e.g., branched-chain amino acids such as leucine, isoleucine, and valine), as well as in lipid emulsification by bile and bile metabolism through bile salt hydrolases (BSHs) and hydroxysteroid dehydrogenases (HSDs), respectively] [137].

Immune system maturation relies on the existence of the microbiota [138]. For example, by adhering to the intestinal epithelium, *Citrobacter rodentium*, a component of the microbiota, stimulates the intestinal production of T helper 17 (Th17) cells and increases the population of intestinal IgA+ cells (plasmatic cells) in the gut lamina propria, and these cells are responsible for producing IgA to maintain homeostasis and protect the mucosa from pathogens and toxins [139]. Commensal bacteria, such as *Akkermansia muciniphila*, significantly influence the temporary maintenance of the optimal parameters of the mucus layer [140]. The intestinal microbial barrier consists of a physically protective layer and produces metabolites, mainly SCFAs (e.g., butyrate), bile acids, and tryptophan derivatives, which strengthen epithelial TJs, influence the cellular response, and inhibit pathogen colonization [141]. Dynamic equilibrium of the microbiota combined with appropriate levels of its metabolites may facilitate epithelial repair, ensuring the integrity of the mucosal lining and preventing the leakage of pathogens and toxins into the bloodstream [142].

Equated with a healthy intestinal microbiome, intestinal health manifests as a variety of physiological functions that go beyond the digestive process and are integrated with the nervous, immune, and hormonal systems to maintain homeostasis of the entire body [143].

**Figure 3:**
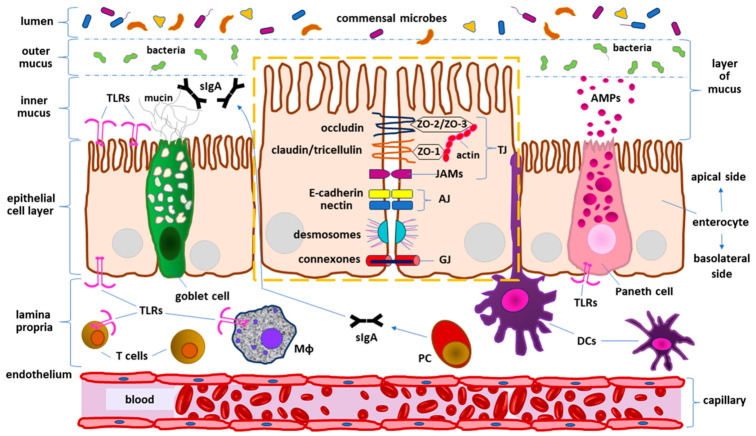
The multi-layered structure of the intestinal barrier [130,133,136,141]. For better clarity, the visual presentation is not to scale. AJ—adherens junction; DCs—dendritic cells; GJ—gap junction; AMPs antimicrobial peptides; JAMs—junctional adhesion molecules; Mφ—macrophage; PC—plasmatic cell; sIgA secretory immunoglobulin A antibodies; TJ—tight junction; TLRs—toll-like receptors; ZO-1, ZO-2, ZO-3–cytoplasmic scaffolding proteins or intracellular adapter (zonula occludens) proteins-1, -2 and -3.

The key structural elements of the intestinal barrier are shown in Figure 3.

Gut microbiota and its metabolites interact with the enteric nervous system (ENS) comprising a network of 200–600 million neurons innervating the GI tract. There is increasing evidence suggesting that bacterial molecules can pass the intestinal epithelial barrier to interact with enteric plexuses directly; they can also act on non-neuronal intermediary cells, whose products can be detected by enteric neurons. Clinical observations confirm that disorders of the ENS correlate with CNS diseases, including NDs. However, the causal relationship between microbial changes and neurological disorders currently remains unproven [144].

### 4.2. A Proinflammatory Diet and Increased Intestinal Permeability 

Abnormally increased intestinal permeability resulting from long-term exposure to proinflammatory dietary factors is the result of several overlapping mechanisms that weaken the intestinal barrier [13]. However, high-fat, high-sugar, or low-fiber diets can also rapidly (within 4 days) shift the microbiome composition, reducing the abundance of beneficial species and promoting inflammation [145]. Key harmful elements of a proinflammatory diet include UPFs that contain emulsifiers, artificial colors, and sweeteners; excess animal fats composed primarily of saturated triglycerides; refined sugars present in white bread, pasta and snacks that promote CLGI; a lack of fiber; and the consumption of alcohol, excessive amounts of caffeine and other stimulants that act as direct irritants of the intestinal mucosa [146].

In response to inflammation, injury, or pathogenic invasion, which characterize an increased intestinal permeability, Paneth cells can experience immediate and complete degranulation, releasing high levels of defensins as an innate immune response to defend against the damaged epithelium [147]. However, the production of dysfunctional defensins (reduced or misfolded) linked to decreased or dysfunctional Paneth cell activity is strongly correlated with intestinal mucosa damage [148]. When the Paneth cell defensin output is not optimal, the resulting dysbiosis allows pathogenic bacteria to thrive, which further breaks down the epithelial barrier and promotes the establishment of a vicious cycle of disease characterized by intestinal leakage [149].

1. **Animal fat**

High levels of animal fat, particularly saturated fat, as well as processed meats and refined sugars in meals, cause a shift in the microbiome that is fundamentally responsible for dysbiosis and characterized by the promotion of the growth of pathobionts (e.g., *Escherichia/Shigella*, *Ruminococcus torques*, and *Bilophila wadsworthia*) while reducing the abundance of beneficial bacteria such as *Akkermansia muciniphila*, *Bifidobacteria* and *Lactobacillus* [150]. The structural integrity of TJ proteins sealing the spaces between adjacent enterocytes is disrupted [150]. The pathomechanisms of an increased intestinal permeability are influenced by both direct toxic effects of nutritional components on intestinal epithelial cells and indirect effects related to alterations in the gut microbiota and increased bile acid production and inflammation [112]. Pathobionts lead to the excessive production of the endotoxin LPS, a major component of the outer membrane of Gram-negative bacteria, which activates TLR4, triggering proinflammatory signaling that degrades TJ proteins [112]. Chronic overconsumption of fat increases the secretion of bile acids, which are then converted by bacteria into toxic, hydrophobic secondary bile acids (e.g., deoxycholic acid). They may stimulate the epidermal growth factor receptor (EGFR) and lead to the dissociation of the occludin–ZO-1 complex, with a subsequent disruption of intercellular junctions [151]. Excess fatty acids are associated with the induction of ROS production in the intestinal epithelium, and the resulting oxidative stress promotes apoptosis; as a result, cell loss increases the formation of free spaces and increases the permeability of the intestinal barrier [151]. By disrupting the unfolded protein response (UPR), a conserved cellular signaling network that maintains protein homeostasis in the endoplasmic reticulum (ER), saturated fats impair mucus production (mainly MUC2) by goblet cells, which allows bacteria to come into direct contact with the epithelial layer, increasing the risk of invasion [11,152].

2. **Refined carbohydrates**

Refined sugar and saturated fat exert synergistic effects on the intestinal barrier. Dysbiosis caused by excess refined sugars in the diet is also accompanied by the reduced expression of TJ proteins [145]. The degradation of junctional proteins is promoted by increased levels of proinflammatory cytokines (e.g., TNF-α and IL-1β) that activate NF-κB and NOD-, LRR- and pyrin domain-containing protein 3 (NLRP3) inflammasomes [153]. Once LPS enters the blood, metabolic endotoxemia occurs that triggers low-grade systemic inflammation. Additionally, an overly porous intestinal lining allows bacteria and undigested food to enter the bloodstream, increasing the inflammatory response, including autoimmune responses [11].

3. **Fiber deficiency**

Diets low in fiber starve the bacteria that produce SCFAs, which are essential for maintaining the normal protective function of the mucus layer within the intestinal barrier [154]. Lack of dietary fiber forces gut bacteria to consume the mucus layer for energy, resulting in a thinning of the physical (mucus) barrier between the intestinal tract and the immune system [154]. Normally, fiber fermentation by beneficial microbes leads to the formation of SCFAs, especially butyrate, which are the primary energy source for enterocytes and are critical for maintaining TJ integrity [155]. Moreover, a low-fiber diet reduces the number of goblet cells and decreases their ability to produce and secrete mucin [156].

**Figure 4:**
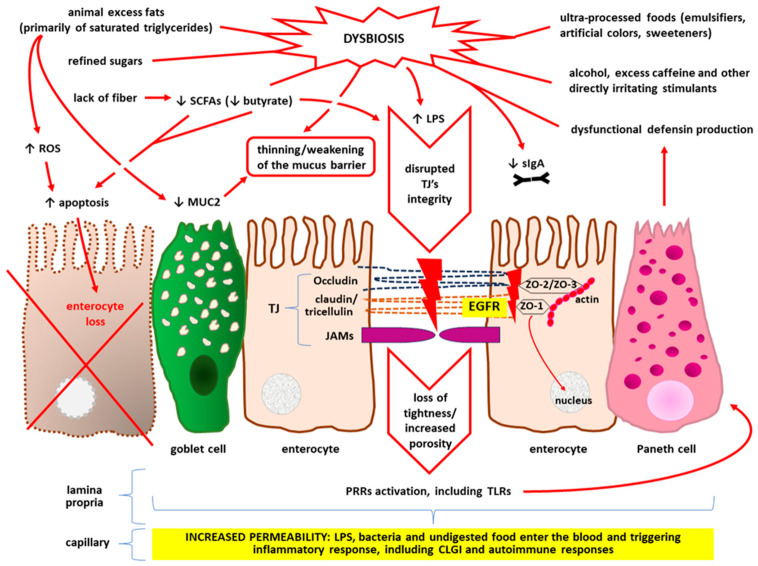
Main overlapping pathomechanisms of abnormally increased intestinal barrier permeability (“leaky gut”) associated with a proinflammatory diet [146,147,148,149]. Dysbiosis occurs, which contributes to the loss of the barrier function of the intestinal epithelium. This leads to disruption of the structural integrity of the tight junction (TJ) proteins (e.g., ZO-1, occludin, claudins) and subsequent loss of tightness between adjacent enterocytes mainly due to pathobionts producing endotoxin (LPS), which triggers proinflammatory signaling via toll-like receptors (TLRs). Excessive bile acids induced by a high-fat diet are a source of toxic hydrophobic secondary bile acids, which activate the epidermal growth factor receptor (EGFR), causing the dissociation and redistribution of zonula occludens-1 (ZO-1) from the cell junction to the cytoplasm or the nucleus. Excessive fatty acids also induce reactive oxygen species (ROS) in the intestinal epithelium, thus promoting apoptosis. Reduced number of enterocytes and their loosened connections in the epithelium result in increased the permeability of the intestinal barrier. Lack of fiber in the diet deprives the body of beneficial microbes that produce short-chain fatty acids (SCFAs, mainly butyrate, critical for maintaining TJ integrity as primary energy source for enterocytes), and causes changes in their metabolism, as they consume the protective mucus layer secreted by goblet cells. Disrupted mucus production, mainly the gel-forming protein mucin2 (MUC2) by goblet cells, allows bacteria to adhere to the epithelial layer, increasing the risk of invasion. The number of goblet cells decreases. In response to loss of tightness/increased porosity of the intestinal barrier, the innate immune system is activated at the level of lamina propria. Chronic stimulation of pattern recognition receptors (PRRs), including TLRs, due to inflammation, injury, or pathogenic invasion, causes defensin disfunction and over production by Paneth cells. A vicious circle mechanism is created where impaired defensin secretion intensifies dysbiosis, facilitating the passage of pathogenic bacteria through the impaired intestinal barrier and maintaining/intensifying the inflammatory response. Secretory immunoglobulin A (sIgA) production, a primary component of the mucosal adaptive (acquired) immune system, also decreases. These responses then spread through the bloodstream as chronic low-grade inflammation (CLGI), including autoimmune responses, when LPS, bacteria, and undigested food enter the blood vessels. JAMs—junctional adhesion molecules; ZO-2, ZO-3—cytoplasmic scaffolding proteins or intracellular adapter (zonula occludens) proteins -2 and -3.

The interdependence of the aforementioned elements of a proinflammatory diet and the pathomechanisms of the development of functional disorders causing increased intestinal permeability are summarized in Figure 4.

## 5. Impact of Long-Term Proinflammatory Dietary Patterns on BBB Integrity

### 5.1. Prerequisites for Normal BBB Function

The BBB plays a key role in CNS homeostasis, involving controlled and regulated isolation from toxins and pathogens while enabling nutrient/waste transport, creating a stable compartment in terms of the ionic balance necessary for efficient neuronal signaling [157]. Interestingly, although the BBB separates the vast majority of CNS structures, circumventricular organs (CVOs), such as the hypothalamus, pineal gland, median eminence, and vomiting center, which monitor the blood composition and regulate autonomic and endocrine functions, are devoid of it [158].

**Figure 5:**
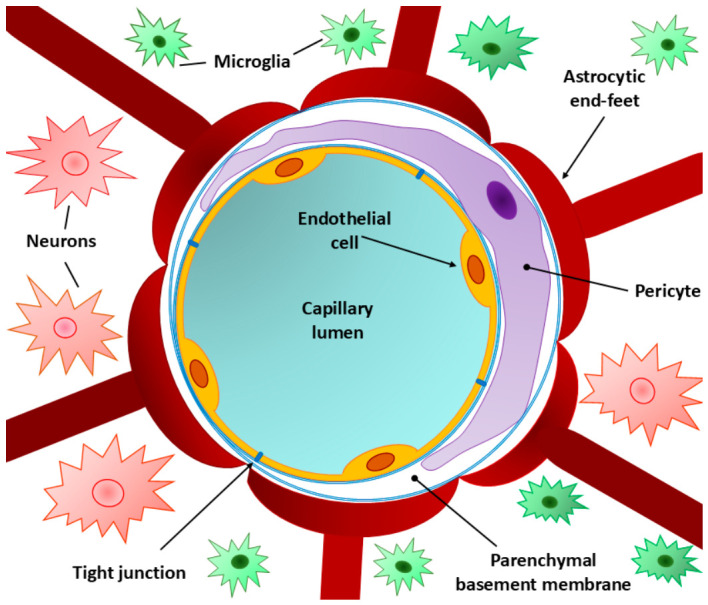
Schematic representation of the blood–brain barrier (BBB) in a cross-section illustrating its cellular counterparts. Adopted from [159], licensed under CC BY 4.0. From the capillary lumen, brain microvascular endothelial cells (BMECs) are surrounded by pericytes located within the parenchymal basement membrane. The presence of tight junctions between endothelial cells maintains the integrity and required level of permeability of the BBB. The surface of the basement membrane is covered by the astrocytic end-feet. Microglia and neuronal synapse endings are located in the extracellular matrix surrounding the BBB from the side of the nervous tissue [160].

The degree of BBB sealing and therefore the degree of restriction of solute and water penetration depend on the functional state of the TJs between the ECs of the capillaries [157]. The protective function against uncontrolled flow into the CNS occurs in several layers: the monolayer of capillary ECs restricts the passage of pathogens (e.g., bacteria, viruses, and fungi) and macromolecules, while the basement membrane ensures the maintenance of barrier integrity and signaling, and the perivascular space (or Virchow–Robin space) stabilizes the structure of the BBB [22,157]. A structural diagram of the BBB is shown in the cross-section in Figure 5.

#### 5.1.1. Brain Microvascular Endothelial Cells (BMECs)

Unlike the capillary endothelium in other regions of the body, which often tends to leak, the capillary endothelium of the BBB consists of tightly connected cells by extensive interendothelial TJs that are devoid of fenestrations [159]. BMECs are more flattened, contain more mitochondria and have fever caveolae, which, by selectively reducing permeability, form a highly impermeable barrier for most hydrophilic macromolecular compounds (including drugs) with high electrical resistance, where the TEER value is often > 1000–3000 Ω/cm^2^ [161]. BMECs exhibit polarization and have distinct luminal (blood-contacting) and abluminal (brain-facing) membranes [162]. Because the paracellular pathway is blocked, molecules must either be lipid soluble (able to cross the membrane directly by passive diffusion, such as O_2_, CO_2_, ethanol, anesthetics, and nicotine) or transported via specific transcellular carrier-mediated systems [e.g., glucose via glucose transporter type 1 (GLUT1), amino acids primarily via the L-type amino acid transporter 1 (LAT1) system and system y+ for cationic amino acids] [163,164]. Water freely crosses the BBB to maintain hydration and balance in the brain tissue via passive diffusion through plasma membranes and through specialized water channels called aquaporins 4 (AQP4), ensuring rapid, continuous exchange. Insulin, transferrin, leptin, and lipoproteins bind to luminal EC surfaces and cross the BBB via receptor-mediated endocytosis (RME) and subsequent transcytosis [165,166]. BMECs further cooperate with the endothelial glycocalyx (ecGCx), a complex, gel-like, carbohydrate-rich meshwork covering the luminal surface of blood vessels, to modulate vascular permeability, immune/inflammatory responses and coagulation within the BBB [167]. Ensuring calcium ion homeostasis within BMECs is crucial for their metabolism, BBB integrity, and signaling related to the inflammatory response and neurovascular coupling (NVC), which enables the adjustment of changes in blood flow to neuronal activity [168].

#### 5.1.2. Endothelial TJs and AJs: Differences from the Intestinal Epithelium

TJs and AJs between BMECs and between enterocytes in the intestine share a similar basic structure and function: they seal the intercellular space and bridge adjacent cells and link to the actin cytoskeleton via catenins respectively [169]. However, the numbers of TJs and AJs differ between ECs and between enterocytes (epithelial cells). While in cell culture, both types may appear similar under an electron microscope, their native functional, structural, and molecular properties are distinct [170].

1. **TJs**

Unlike epithelial TJs in the intestine, endothelial TJs are often less structured, more variable, and intermixed with AJs along the lateral membrane rather than being strictly apical [169]. In endothelial TJs, claudin-5 is more frequently the main component, and the regulation of permeability and leukocyte trafficking is strongly dependent on junctional adhesion molecules (JAMs) [171]. Both the TJs between BMECs and within intestinal epithelium are generally tight and sealed. The absence of desmosomes, the presence of which is a characteristic of epithelial tissues, distinguishes endothelial TJs not only within the BBB but also in other locations [170].

2. **AJs**

The key differences between AJs between BMECs and those in the intestinal epithelium concern the major transmembrane proteins. Adhesion between BMECs is mediated by vascular endothelial (VE)-cadherin (MW approx. 130 kDa), whereas enterocytes use E-cadherin (MW approx. 140 kDa) as the primary adhesion molecule to form a stable belt-like structure [172,173]. VE-cadherin (CD144, cadherin-5) is unique to the endothelium and has more specialized functions, as it is essential for the formation of vascular structures, maintains the integrity of the vascular wall, and acts as a mechanoreceptor [174]. The absence of desmosomes between ECs results in the specific molecular composition of AJs, where VE-cadherin directly associates with β-catenin and α-catenin [170]. Compared with enterocytes, ECs often form “discontinuous” or “punctate” AJs that are more dynamic, allowing for increased turnover to manage leukocyte migration and vascular permeability [175].

**Figure 6:**
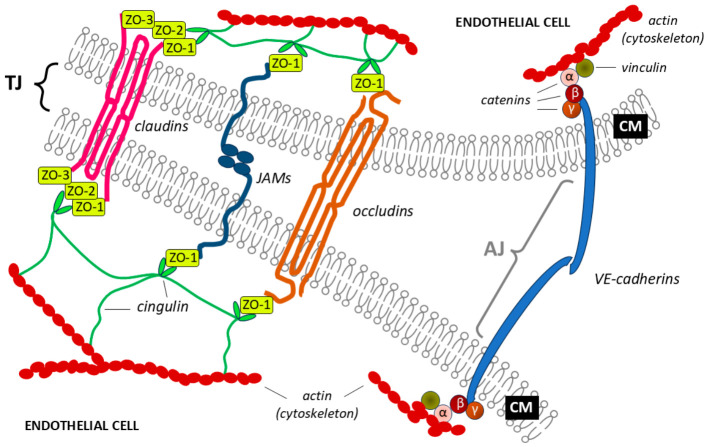
Schematic representation of the tight contact between adjacent endothelial cells. Adopted from [159], licensed under CC BY 4.0. Tight junctions (TJs) are formed by the joining of major cellular membrane (CM) proteins of adjacent cells, such as claudins, occludins, and junctional adhesion molecules (JAMs). Additional cytoplasmic proteins of adjacent cells participate in tight junction stabilization by binding to elements of the cytoskeleton (actin) of endotheliocytes. These cytoplasmic proteins include zonula occludens-1, -2, and -3 (ZO-1, ZO-2, and ZO-3, respectively); cingulin; vinculin; and catenins (α, β, and γ). A much weaker adherens junction (AJ) connects the adjoining plasma membranes with the electron-dense fibrillary molecules known as vascular endothelial (VE)-cadherins [22].

The main structural elements and organization of endothelial TJs and AJs are shown in Figure 6.

#### 5.1.3. Pericytes

Located outside the capillary endothelium and sharing a basement membrane, pericytes are crucial perivascular cells in the BBB that envelop BMECs to maintain structural and functional integrity [176]. Pericytes regulate BBB permeability, support BMECs and promote the formation of TJs, influencing claudin-5 and occludin expression. A lack or dysfunction of pericytes leads to increased vesicular transport (transcytosis) and breakdown of the barrier. Due to their macrophage-like phagocytic properties, pericytes are also essential for removing toxic metabolites and cellular byproducts from the microcirculation, particularly within the CNS, where they clear neurotoxins, such as amyloid-beta (Aβ) [177]. Pericytes express BBB-specific genes and receptors for vasopressin, angiotensin, and endothelin, which, combined with their contractile properties, enable them to participate in the cerebral autoregulation of blood flow by controlling the capillary diameter in accordance with the metabolic demands of neurons [178,179]. Pericytes possess stem cell-like properties and participate in processes related to injury repair by differentiating into other cell types [180]. They are also essential for regulating vascular development through the release of vascular endothelial growth factor (VEGF) and angiopoietin-1 (Ang-1) and stabilizing new vessels by regulating basement membrane formation through the secretion of extracellular matrix (ECM) proteins [181]. Finally, pericytes act as sensors of inflammation, regulating the recruitment and transmigration of leukocytes across the BBB [176,182]. The loss of pericytes can trigger uncontrolled inflammatory responses within the CNS [182]. The heterogeneity of pericytes motivates intensive research aimed at the precise identification of pericyte subsets in humans, which could be promising targets for planning therapies for various NDs [183].

#### 5.1.4. Basement Membrane

The basement membrane (BM) of capillaries within the human BBB is a 50–100 nm thick, organized ECM sheet composed primarily of collagen type IV, laminins 211/411/511, nidogens (essential glycoprotein components of the BBB, also known as entactins), agrin and perlecan, the major heparan sulfate proteoglycans (HSPGs) of BMs [184,185]. BM components are secreted mainly by ECs, pericytes and astrocytes and are arranged in a double-layered structure, which includes an inner vascular BM (surrounding ECs and pericytes) and an outer parenchymal BM (associated with astrocyte endfeet) [160]. The BM acts as a dynamic scaffold that stabilizes the BBB and anchors cells (ECs, pericytes, and astrocytes), and, by regulating the passage of molecules, it also acts as a selective barrier, including signal transduction [186]. The thickening of the BM is typically associated with brain pathology and neurodegeneration observed in aging and various neuropathological conditions characterized by the accumulation of ECM proteins (such as collagen IV) [187,188]. However, thinning through the degradation of BM driven by matrix metalloproteinases (MMPs) in inflammation, including CLGI, leads to neurodegeneration due to the detachment of astrocytic endfeet, increased permeability, and increased leukocyte infiltration [189].

#### 5.1.5. Perivascular Membrane

The perivascular membrane of the BBB, often referred to as parenchymal BM or glia limitans perivascularis, constitutes the outer layer formed primarily by specialized astrocyte processes (endfeet), which separates the vessels from the brain parenchyma proper [190]. Astrocytes not only form the perivascular membrane but also modulate the expression of TJ proteins of ECs through the expression of VEGF and angiotensin-1 [186,191]. Neuroinflammation has been shown to be associated with BBB perivascular membrane dysfunction [189].

Assessing the effects of increased concentrations of proinflammatory factors (e.g., cytokines) carried in the blood on the integrity of the BBB requires a detailed understanding of the structure, function and interaction of all the aforementioned BBB components [192].

### 5.2. Diet-Derived or Diet-Related Proinflammatory Factors in the Blood and BBB Dysfunction

The long-term presence of elevated levels of CLGI markers, including those caused by the loss of intestinal barrier integrity due to the use of proinflammatory diets, is accompanied by BBB dysfunction [193]. The pathomechanism of the development of disorders of BBB structure and function in CLGI is well reflected in the analysis of the activity of proinflammatory cytokines, among which IL-6 and TNF-α are key biomarkers of CLGI and, consequently, are markers of NDs [193].

In terms of the structure of the BBB, two mechanisms of cytokine penetration can be distinguished: nondisruptive and disruptive [159]. The clinical significance of cytokine penetration through the BBB hinges on whether the process is disruptive (anatomical damage) or nondisruptive (regulated transport). Cytokines penetrate the BBB in NDs through both nondisruptive and disruptive mechanisms, with diet-induced CLGI accelerating the shift toward disruptive, high-permeability pathways [1,159,193].

#### 5.2.1. Nondisruptive Crossing

Rapid passage (within 30 min to a few hours) without structural damage to the BBB occurs when certain proinflammatory cytokines (e.g., IL-1α, IL-6 and TNF-α), most often of exogenous origin, cross the BBB after an intravenous injection. This passage is achieved by retrograde axonal transport, saturated influx transport (SIT), and by simple diffusion in brain areas where the BBB is incomplete [158,194,195]. SIT refers to a phenomenon that is often described by Michaelis–Menten kinetics, where the rate of a substance (e.g., cytokine) entering a cell or tissue reaches a maximum velocity (V_max_) and cannot increase further because all available membrane carrier proteins or transporters are occupied [196].

The compromised function of the barrier may be caused by the activation of cytoplasmic free calcium, which can disturb homeostasis in the brain [195]. Ca^2+^ ions released from cellular organelles into the cytoplasm are key intracellular messengers in many cell types, including BMECs, where they trigger secretory exocytosis [197].

#### 5.2.2. Disruptive Crossing

When chronically elevated levels of endogenous proinflammatory cytokines lead to TJs damage with the degradation of membrane proteins and promote immune cell infiltration, the strictly selective barrier function of the BBB between the bloodstream and the CNS is severely disrupted [192,194]. Proinflammatory cytokines not only pass through the BBB but also actively increase its permeability, creating a vicious cycle of further infiltration [195]. Disruptive crossing of cytokines through the BBB is a critical mechanism of neuroinflammation, where peripheral inflammatory signals enter the cerebral compartment. Consequently, the facilitated access of proinflammatory cytokines (including chemokines), neurotoxins, pathogens and inflammatory cells to the CNS drives/sustains neuroinflammation and is associated with the development of NDs (e.g., AD and PD) and psychiatric disorders (e.g., depression and anxiety) [192,198].

The interaction between the gut barrier and the BBB is a key element of the so-called gut–brain axis [199]. Thus, a disruption in the intestinal barrier (the so-called “leaky gut”) due to a diet characterized by high DII scores (a proinflammatory diet) initiates a cascade of events that directly threaten the structural integrity of the BBB and cause disruptive crossing of cytokines through this barrier toward the brain [19,200].

**Figure 7:**
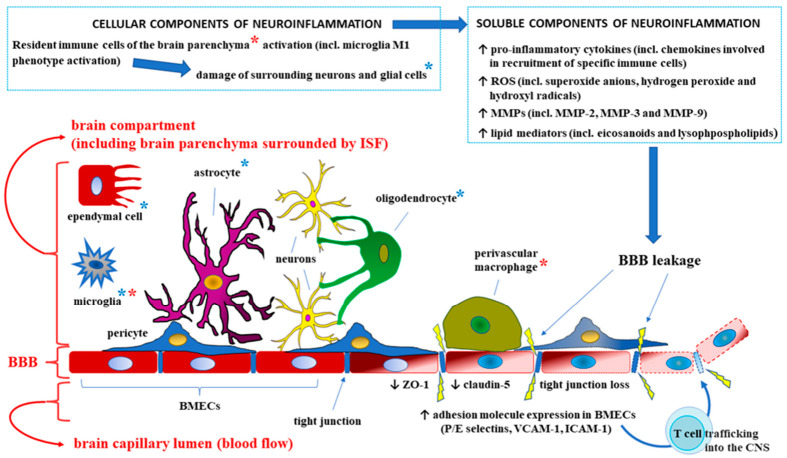
The endogenous pathway of proinflammatory cytokines through the blood–brain barrier (BBB). Adopted from [159], licensed under CC BY 4.0. Neuroinflammation may lead to decreased BBB integrity due to the activation of brain-resident immune cells (marked with red asterisks), which then results in the damage of surrounding neurons and glial cells (marked with blue asterisks). The cellular components of neuroinflammation (summarized in the box on the left) are accompanied by soluble components, such as overproduced proinflammatory cytokines and other inflammatory mediators (listed in the box on the right) in inflammation-activated cells (e.g., in polarized states towards a proinflammatory (M1) phenotype microglia). Decreased expression of membrane proteins (e.g., claudin-5) and cytoplasmic proteins (e.g., zonula occludens-1 (ZO-1)) is accompanied by the loosening of intercellular tight junctions. Combined with an increase in the expression of adhesion molecules on the surface of the brain microvascular endothelial cells (BMECs), an increase in BBB permeability may result in the influx of T cells from the bloodstream into the central nervous system (CNS) [201,202]. Other abbreviations: ROS—reactive oxygen species; MMPs—matrix metalloproteases.

BBB damage and the development of neuroinflammation mediated by the mechanism of disruptive crossing of endogenous proinflammatory cytokines are shown in Figure 7.

### 5.3. Biomarkers of BBB Integrity

Loss of BBB integrity can be detected in the clinic by assessing the concentrations of biomarkers in blood and CSF and by performing biomarker-based imaging studies [203].

#### 5.3.1. Fluid-Based Biomarkers

1. **Q-Alb**

The most widely used fluid biomarker of BBB function/dysfunction is the cerebrospinal fluid (CSF)/serum albumin ratio, which is also known as the albumin quotient (Q-Alb) [203]. Since albumin is not produced in CSF, its presence is a consequence of BBB permeability. Hence, the CSF/serum albumin ratio [Q-Alb = CSF albumin (mg/dL)/serum albumin (g/dL) × 10^−3^] is a key diagnostic marker for BBB integrity, with higher values indicating increased permeability. The normal range of Q-Alb values is, depending on age, ≤ 5.0 x 10^−3^ (<15 years), < 6.5 (15–40 years), and < 8.0 (41–60 years) to < 9.0 (>60 years). A commonly used age-adjusted reference for the upper limit is the so-called Reiber formula: 4 + (age/15) [204,205]. The median Q-Alb values also show gender differences, reaching higher values in men than in women within the same age groups. Furthermore, for a given patient, the Q-Alb value may need to be corrected to take into account the individual blood concentration of albumin [205,206].

While exceeding the above age-adjusted thresholds is considered initially indicative of BBB dysfunction, clearly pathological (age-independent) thresholds in the range of values 10 < Q-Alb ≤ 30 indicate a moderate impairment, and >30 indicates a severe impairment of BBB integrity [207]. An abnormally elevated Q-Alb is associated with a variety of neurological disorders, including both inflammatory and noninflammatory disorders [206]. Mild-to-moderate increases in Q-Alb are typically observed in patients with viral meningitis and polyneuropathy due to diabetes and immune diseases, whereas moderate-to-severe Q-Alb elevations are associated with severe CNS infections, severe spinal canal stenosis and immune-mediated polyradiculitis [208,209]. The results are generally reliable, although CSF albumin levels may be influenced by altered proteolytic activity [204].

2. **Q-IgG**

Often used in conjunction with Q-Alb is the immunoglobulin G quotient (Q-IgG) or the CSF/serum ratio of IgG. Q-IgG reflects both the rate of IgG permeability into the CSF from the blood (leakage) and the intrathecal synthesis of IgG (production within the CNS) [210].

If the levels of both Q-Alb and Q-IgG are elevated, they suggest a damaged BBB that can leak. If the level of Q-IgG is higher than expected based on the Q-Alb (often interpreted using Reiber’s formula or a Reibergram), it indicates active local (intrathecal) IgG synthesis, which is typically observed in patients with multiple sclerosis [211]. Therefore, Q-Alb is typically considered a more direct measure of global BBB permeability, whereas Q-IgG is particularly essential for detecting immunoglobulin-mediated immune responses (e.g., oligoclonal bands) [212].

3. **Other biomarkers**

Other newer and more targeted biomarkers of BBB integrity whose usefulness in the diagnosis of specific neuroinflammatory diseases and NDs that are still being validated include the following:

– Platelet-derived growth factor receptor-β (PDGFRβ), which is released during pericyte injury [213];
– Angiopoietin-2 (Ang-2), which is released from ECs in response to hypoxia and oxidative stress [214];
– Glycoprotein YKL-40 [also known as chitinase-3-like protein 1 (CHI3L1) or human cartilage glycoprotein 39 (HC gp-39)], which is abundantly expressed in reactive astrocytes during neuroinflammation [215];
– Vascular endothelial cadherin (VE-cadherin), a calcium-dependent cell adhesion receptor expressed exclusively on ECs with elevated cerebral expression that is indicative of cognitively unimpaired patients with preclinical AD [216];
– AQP4, a water channel that is expressed mainly in astrocytic endfeet, whose dysregulation in the brain has been shown to be associated with NDs and whose concentrations positively correlate with the severity of BBB dysfunction (as defined by Q-Alb) and CSF biomarkers of tau pathology (e.g., p-tau181, p-tau217, and p-tau231) [217].

#### 5.3.2. Imaging-Based Biomarkers

Medical imaging techniques, including magnetic resonance imaging (MRI) and positron emission tomography (PET), are used to assess BBB integrity [203].

1. **MRI**

Both contrast-enhanced and noncontrast-enhanced MRI techniques are used to quantitatively assess BBB regional permeability. In contrast-enhanced methods, gadolinium-based contrast agents (GBCAs), whose molecular size is too large (∼559 to 1058 Da) to cross an intact BBB, are administered intravenously [218]. Damage to junctional complexes within the endothelium, such as TJs, AJs, and desmosomes, leads to the flow of GBCAs into the brain compartment via paracellular transport [219]. Noncontrast methods measure permeability using magnetically labeled water molecules. Calculations of BBB integrity using noncontrast-based techniques take into account that water has a small molecular size (∼18 Da) and can pass through the BBB paracellularly or through transcellular mechanisms of active cotransport and free diffusion across the endothelial membrane and facilitates diffusion through AQP4 water channels expressed in astrocytic endfeet [220,221].

2. **PET**

PET is used to measure the radioactive decay of positron-emitting radionuclides (radioisotopes) used as radiotracers or radiopharmaceuticals, such as ^11^C, ^18^F, ^13^N and ^15^O [222]. These radiotracers decay by emitting positrons, which interact with electrons to produce 511 keV gamma rays, allowing scanners to image metabolic activity and the tracer concentration in tissue [223]. In practice, PET imaging assesses BBB integrity by noninvasively measuring the rate at which a specific radiopharmaceutical, such as (R)-[^11^C]verapamil, leaks from blood vessels into brain tissue [223,224]. Unlike MRI, which is often used to visualize large-scale structural damage, PET provides molecular-level insights into BBB disruption, transporter dysfunction (e.g., P-glycoprotein), and permeability-surface area (PS) product changes associated with selected disease states, including AD, PD, epilepsy, and tumors [224].

## 6. Diet-Dependent Proinflammatory Signaling via the Gut–Brain Axis and Neurodegenerative Diseases

The gut hosts 70–80% of the immune cells in the body, primarily within the lamina propria and epithelial layer, making it the largest immunological organ [225]. This high concentration of immune system cells, including IgA-producing B cells and T cells, acts as a primary defense barrier and is fundamental to maintain intestinal homeostasis [226]. Obtaining immunocompetence by these cells to quickly distinguish between harmless antigens and pathogens, as well as to generate an adequate and effective immune response against the latter while maintaining tolerance to food and beneficial bacteria, is crucial [227]. The gut microbiota trains the cells of the immune system in this area from the first days of extrauterine life [228]. Microbial colonization begins immediately after birth, with infants acquiring bacteria from their mothers (vaginal, feces, skin, and milk) and the surrounding environment, typically maturing into a stable, adult-like composition by the end of the first 3 to 5 years of life. This process represents a critical window of development, often referred to as the first 1000 days, during which the initial, highly variable microbial community matures and is influenced heavily by factors such as the delivery mode, feeding habits, and environment [229].

Since the intestines are the main source of inflammatory signals within the gut–brain axis, which are part of a bidirectional communication pathway, dysbiosis or a microbial imbalance may trigger systemic inflammation, typically CLGI, that impacts the CNS [225]. An inappropriate diet, which is reflected in a high DII score, leads to disturbances in the intestinal microbiome and, consequently, a loss of intestinal barrier integrity, with the prospect of a destructive effect of increased concentrations of blood-borne proinflammatory factors on the BBB [18,192,230]. Thus, the diet influences the risk of noncommunicable inflammatory diseases, including NDs [11,231]. Several NDs are linked to chronic neuroinflammation, which is frequently exacerbated by a proinflammatory diet, and high DII scores are correlated with a higher risk of neurodegeneration [12,23,232]. In order to remain critical when interpreting data on individual NDs, it is worth remembering that due to the lack of other diet-(DII score)-oriented studies, the focus was limited to the presence of neuroinflammation in these diseases. Therefore, by necessity, this review is an extrapolation from CLGI data to DII-specific risk. Data concerning the participation of proinflammatory factors, the presence of inflammatory markers and changes in the activity of immune cells in common NDs are summarized in Table 2, Table 3 and Table 4, respectively.

The relevance of research investigating the relationship between DII score, CLGI and NDs is confirmed by growing number of recently published research papers. However, the analysis of Table 2, Table 3 and Table 4 shows that there are no publications in the literature presenting the results of studies that would directly relate the DII score to the population of patients with NDs. An exception is the prospective cohort study published by Shi et al., 2023 [232], which analyzed a total of 166,377 UK Biobank participants without dementia at baseline. The correlation between DII score and the risk of different subtypes of dementia, including Alzheimer’s dementia and vascular dementia, was investigated. Given the changes in brain structure observed in patients already suffering from dementia, the association between DII and magnetic resonance imaging (MRI) findings of brain structure were also investigated, potentially revealing the mechanisms linking a pro-inflammatory diet to cognitive decline. During a median follow-up time of 9.46 years, a total of 1372 participants developed dementia. It was shown that the incidence of all-cause dementia increased by 4.6% for each additional unit of DII. In addition, DII displayed a “J-shaped” non-linear association with Alzheimer’s dementia (P_nonlinear_ = 0.003). For DII scores above 1.30, an increase in DII was significantly associated with an increased risk of Alzheimer’s dementia. In brain MRI, the total volume of white matter hyperintensities increased with an increase in DII, whereas the volume of gray matter in the hippocampus decreased. The authors concluded that higher DII was associated with a higher risk of all-cause dementia and Alzheimer’s dementia, while the association of DII with vascular and frontotemporal dementia was not significant [232].

In another cohort epidemiological study, a total of 11,957 community residents aged 65 years or older were recruited in eight sites in Japan (JPSC-AD Study) [238]. The relationships of serum hs-CRP with dementia including AD and with regions of interest of brain MRI were investigated. Multivariable-adjusted odds ratios (ORs) for all-cause dementia and for AD prevalence increased significantly with higher serum hs-CRP levels (*p* for trend < 0.001 and *p* = 0.001, respectively). In addition, results from 8614 individuals in the same cohort who underwent brain MRI revealed that the multivariable-adjusted temporal cortex volume/estimated total intracranial volume ratio decreased significantly with increasing serum hs-CRP levels (<1.0 mg/L 4.28%, 1.0–1.9 mg/L 4.27%, 2.0–2.9 mg/L 4.29%, ≥ 3.0 mg/L 4.21%; *p* for trend = 0.004). The authors conclude that elevated serum hs-CRP levels are associated with greater risk of presence of dementia, especially AD, and of temporal cortex atrophy in a community-dwelling Japanese older population [238].

It is particularly important to take DII into account when developing a diet for people with genetic predispositions to neurodegenerative diseases, aimed primarily at delaying the onset of symptoms, reducing inflammation and protecting neurons from oxidative stress [353]. This applies, for example, to people with the *APOE* ε4 allele—the strongest genetic risk factor for late-onset AD, LRRK2 gene mutations—a leading genetic cause of familial and sporadic PD, as well as C9orf72 mutations, (specifically a GGGGCC hexanucleotide repeat expansion)—the most common genetic cause of amyotrophic lateral sclerosis (ALS) and frontotemporal dementia (FTD) [354,355,356,357]. Characterized by a low DII score, the MIND diet (Mediterranean-DASH Intervention for Neurodegenerative Delay) is specifically designed to protect brain health and reduce the risk of neurodegenerative diseases, such as AD, by combining elements of the Mediterranean and DASH (Dietary Approaches to Stop Hypertension) diets. Studies suggest it is highly effective for individuals with a genetic predisposition to dementia, including those with the APOE-ε4 allele, as high adherence can help mitigate this risk [358,359,360].

The meaning of the same DII scores in the context of NDs varies depending on sex and age [361]. These two factors act as critical, independent modifiers that may determine how a proinflammatory diet impacts the brain, to what extent it is accelerating neurodegenerative processes and cognitive decline, particularly in aging. At puberty, sex differences in gut microbiota become particularly pronounced, with females generally exhibiting higher alpha diversity (greater number of species) and a higher *Firmicutes*-to-*Bacteroidetes* ratio than males. Women had higher amounts of *Akkermansia* and Bifidobacterium compared to men, while males showed higher levels of *Prevotella* [362]. Immune system function, including neuroimmune function, is also subject to sex differences through hormonal, genetic, and epigenetic mechanisms. Females generally mount stronger innate and adaptive immune responses than males but are more susceptible to autoimmune diseases [363]. Moreover, while the aging process itself induces profound alterations in central and systemic immune regulation, leading to CLGI, immune aging (immunosenescence) manifests differently between sexes [364,365]. Generally, men, with age, experience a more rapid decline in naive T cells, decreased CD4+/CD8+ ratios, and increased accumulation of senescent T cells, contributing to diminished adaptive immunity [366,367]. Women, on the other hand, retain more effective immune surveillance for longer but also exhibit higher levels of CLGI or inflammaging [368]. The hormonal shifts that accompany menopause and andropause further modulate immunosenescence. Decrease in estrogen levels after menopause is associated with increased systemic inflammation and decreased neuroprotection, while decline in testosterone in men have been linked to diminished immunomodulation and increased susceptibility to neuroinflammation [369,370]. Interpretation of the DII score must therefore take into account the different functioning of the microbiota–gut–brain axis depending on the sex and age of the individual [371]. To demonstrate a direct relationship between DII score and BBB integrity, future BBB permeability studies (e.g., using Q-Alb, Q-IgG, or imaging-based) in humans stratified by DII are necessary. It should be noted that while the DII is designed exclusively for evaluating the human diet, a significant proportion of the experimental data on the effects of specific diets on CLGI, intestinal barrier integrity, BBB permeability, and NDs comes from animal studies, primarily rodent models, therefore direct translation of these data to humans is difficult.

When discussing the potential importance of high DII scores in the pathomechanism of NDs, it should be taken into account that the gut–brain axis is a bidirectional communication network linking the CNS (brain) and the ENS (gut), which enables constant dynamic signaling via neural, but also via immune and endocrine pathways [372,373]. Therefore, proinflammatory diet patterns may disrupt gut microbiota, which communicates with the brain via afferent pathways (gut-to-brain communication) via metabolites, immune system modulation, and neurotransmitters like serotonin (5-HT) and dopamine. Gut microbiota synthesizes and modulate key neurotransmitter precursors, notably 5-HT and gamma-aminobutyric acid (GABA), influencing gut–brain communication via the gut–brain axis. Specific microbes (e.g., *Lactobacillus*, *Bifidobacterium*) produce GABA, while others (e.g., spore-formers) stimulate enterochromaffin cells to produce 5-HT from tryptophan [374]. On the other hand, the existence of an efferent pathway (brain-to-gut communication) causes the brain to act on the gut through the autonomic nervous system, the hypothalamic–pituitary–adrenal (HPA) axis and neurotransmitter release, influencing intestinal motility, permeability, and blood circulation [372]. Therefore, such bidirectional communication enables gut microbiota to influence brain functions and emotions, while allowing the brain regulate gut physiology [373]. Further research is needed to clarify whether NDs may involve a vicious cycle mechanism in which efferent signaling from CNS leads to disruption of the intestinal barrier integrity [144].

High-adherence dietary interventions, especially those based on low DII score diets such as the MIND and Mediterranean diets, are highly effective in reducing the risk and slowing the progression of NDs [375,376,377,378]. High-adherence dietary interventions, especially those based on low DII score diets such as the MIND and Mediterranean diets, are highly effective in reducing the risk and slowing the progression of NDs. In one study, researchers found a 53% lower rate of Alzheimer’s disease for those with the highest MIND diet scores (indicating a higher intake of foods on the MIND diet). Even those participants who had moderate MIND diet scores showed a 35% lower rate compared with those with the lowest MIND scores. [379]. Several clinical studies have indicated benefits of the Mediterranean diet in neurodegenerative conditions, including MS (NCT05175378), PD (NCT03851861), and AD (NCT04435509 and NCT04440020) [380]. These diets (the MIND and mediterranean), along with calorie restriction and flavonoid-rich nutrition, work by mitigating oxidative stress, reducing neuroinflammation, and enhancing cognitive resilience. Omega-3 fatty acid supplementation is also beneficial, reducing oxidative stress and neuroinflammation and supporting neuronal membrane integrity. Evidence suggests that omega-3 supplementation protects against diseases like AD and PD and can also delay cognitive decline in early AD’s stages [381,382]. Importantly, the use of both diets with a low DII score is associated with a reduction in the concentrations of CLGI markers in blood, improvement of intestinal microbiota with an increase in microbial diversity and strengthening the integrity of both the intestinal barrier and the BBB [383,384,385,386,387]. During dietary treatment, probiotics (live microorganisms), prebiotics (dietary fibers), and synbiotics (a combination of prebiotics and symbiotics) should be introduced because they are effective in regulating gut microbiota and enhancing intestinal permeability by promoting beneficial bacterial growth and reducing inflammation [388]. It is also important to remember that UPFs consumption is a primary driver of high DII scores in modern Western populations, significantly contributing to CLGI [389]. Diets rich in UPFs—such as sweetened drinks, packaged snacks, and ready meals—now account for over 55% of daily energy intake in the US and UK, directly correlating with elevated inflammatory markers like C-reactive protein (CRP) [389,390]. Evidence strongly suggests that UPFs should be minimized or avoided in a diet focused on preventing NDs [391].

In recent years, CLGI has been shown to play a role not only in the development of NDs, but also in the pathomechanisms of psychiatric disorders (e.g., depression, anxiety, psychosis) [392,393]. Although NDs and psychiatric disorders differ primarily in their underlying pathology—progressive irreversible structural damage versus functional, neurochemical imbalances which may not show early structural changes—they are united by an overlapping mechanism, which includes post-translational modifications, the microbiota–gut–brain axis, and signaling events [394,395]. However, the intention of the authors of this paper was to address the role of CLGI only in NDs.

## 7. Concluding Remarks

The type of diet and eating habits directly determine long-term health and are critical risk factors for development of chronic diseases. The proinflammatory potential of the diet, as expressed by the DII score, may be associated with the risk of gut microbiome disorders and loss of intestinal barrier integrity (increased intestinal permeability) and/or with BBB dysfunction in the case of NDs. The exact, holistic pathomechanisms of common NDs (AD, PD, ALS, MS and HD) are not fully understood, but the common factor underlying irreversible damage to cognitive, motor, and mental functions is neuroinflammation, developing in the background of resoleomic disorders and CLGI.

In this paper, we performed a comprehensive review of the key components that may play an important role in the pathomechanisms of NDs in relation to the proinflammatory potential of the diet expressed by the DII score. 

The parallel presentation of the intestinal barrier, systemic inflammation (CLGI) with its biomarkers in blood and the BBB as three compartments/demarcations (intestinal, blood and brain) was intentional. This is due to the fact that the interdependencies between these compartments in the NDs are not obvious, due to the large number of variables influencing the inflammatory process and the lack of precise in vivo research models.

Highlighting the importance of the DII score as a potential risk indicator for NDs or as a key parameter in dietary treatment for NDs is innovative compared to the existing literature and may constitute a starting point for preparing a systematic review on this topic.

The co-occurrence of elevated levels of markers of low-grade inflammatory response in blood, associated with intestinal leakage and corresponding to CLGI, and elevated biomarkers of BBB dysfunction provide evidence that diet-dependent proinflammatory signaling via the gut–brain axis is important for the development of NDs. Consequently, an indirect relationship between DII and NDs can be demonstrated by quoting the results of studies in which high-adherence dietary interventions, using low DII score-based diets such as the MIND and Mediterranean, were effective in reducing the risk or slowing the progression of NDs.

However, the authors are aware that the interpretation of the available data is not free from limitations, resulting primarily from the lack of publications on cohort epidemiological studies that selectively/directly consider the relationship between DII score and NDs, instead of referring to the role of diet in the development of CLGI and neuroinflammation. In this review, we extrapolated from CLGI data to DII-specific risk, also being aware of a recognized need to improve DII calculation methods to enhance accuracy, strengthen evidence-based links between diet and inflammation, and improve clinical applicability.

In summary, the development of an individualized diet in terms of nutritional and caloric value while maintaining its anti-inflammatory properties, as assessed by the DII score, should be a prerequisite for specialized causal pharmacological treatment of individual NDs to the extent possible. Future research should be aimed at improving the DII and demonstrating in prospective studies on sufficiently large groups of patients whether the DII score can be an independent indicator for assessing the risk of NDs and/or potential therapeutic effects. Current knowledge does not provide evidence to confirm or rule out this possibility. Another important issue is the standardization of CLGI biomarkers for clinical applications that would ensure possible comparisons between studies and different cohorts addressed.

## Figures and Tables

**Table 2:** Blood biomarkers of chronic low-grade inflammation (CLGI) in common neurodegenerative diseases.

Neurodegenerative Disease	Biomarkers of CLGI in Blood				Ref.
	TNF-α	IL-1	IL-6	hsCRP	
Alzheimer’s Disease (AD)					[233,234,235,236,237,238,239,240]
Parkinson’s Disease (PD)					[241,242,243,244,245]
Multiple Sclerosis (MS)				 *	[246,247,248,249,250,251]
Amyotrophic Lateral Sclerosis (ALS)					[252,253,254,255,256,257,258,259]
Huntington’s Disease (HD)					[260,261,262,263,264]

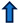
—increased; 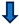
—decreased; 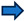
—no change; TNF-α—tumor necrosis factor alpha; IL-1—interleukin 1; IL-6—interleukin 6; hsCRP—high sensitivity C-reactive protein; Ref.—references. * in active stage of MS.

**Table 3:** Biomarkers of blood–brain barrier (BBB) integrity in common neurodegenerative diseases.

Neurodegenerative Disease	Biomarkers of BBB Integrity			Ref.
	Q-Alb	Q-IgG	Other *	
Alzheimer’s Disease (AD)	INC ( 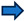 , 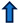 )	INC ( 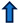 , 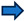 , 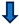 )	 PDGFRβ  Ang-2  YKL-40  AQP4  VE-cadherin	[265,266,267,268,269,270,271,272,273,274,275,276,277,278]
Parkinson’s Disease (PD)			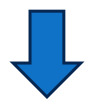 PDGFRβ 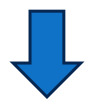 Ang-2  YKL-40 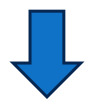 AQP4 ND for VE-cadherin	[216,279,280,281,282,283,284,285,286,287,288,289]
Multiple Sclerosis (MS)	 CNS damage; 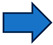 no CNS damage		 PDGFRβ  Ang-2  YKL-40 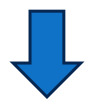 VE-cadherin INC for AQP4 ( 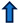 , 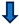 )	[209,290,291,292,293,294,295,296,297]
Amyotrophic Lateral Sclerosis (ALS)			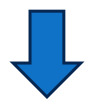 PDGFRβ  Ang-2  YKL-40  AQP4 INC for VE-cadherin ( 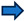 , 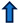 )	[298,299,300,301,302,303,304,305,306,307,308]
Huntington’s Disease (HD)			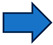 PDGFRβ  Ang-2  YKL-40 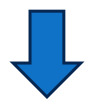 AQP4 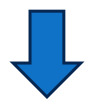 VE-cadherin	[309,310,311,312,313,314,315,316,317,318]

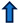
—increased; 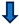
—decreased; 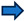
—no change; INC—inconsistent; ND—no data; Q-Alb—albumin quotient; Q-IgG—immunoglobulin G quotient; * other biomarkers of BBB integrity: PDGFRβ—platelet-derived growth factor receptor-β; Ang-2—angiopoietin 2; YKL-40—glycoprotein YKL-40 [also known as chitinase-3-like protein 1 (CHI3L1) or human cartilage glycoprotein 39 (HC gp-39)]; AQP4—aquaporin 4 (water channel); VE-cadherin—vascular endothelial cadherin; CNS—central nervous system; Ref.—references.

**Table 4:** Immune cell activity in common neurodegenerative diseases. 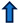
—increased; 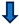
—decreased; 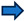
—no change; CONT—contradictory; Mono—monocytes; Mφ—macrophages; M1—pro-inflammatory polarized macrophages; DCs—dendritic cells; CD4^+^ T cells, or helper T cells; CD8^+^ T cells or cytotoxic T lymphocytes; T_reg_—regulatory T cells; Th17.1.—a subset of highly pathogenic CD4^+^ T helper cells; NK cells—natural killer cells; Ref.—references.

Neurodegenerative Disease	Activity of Immune Cells in Peripheral Blood or Brain Tissue (Mφ)					Ref.
	Mono	Mφ	DCs	T Cells	B Cells	
Alzheimer’s Disease (AD)			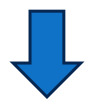	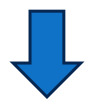 Treg	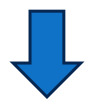	[237,319,320,321,322,323,324,325]
Parkinson’s Disease (PD)			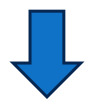	 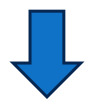 Treg	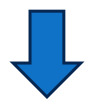	[326,327,328,329,330,331,332,333,334,335,336]
Multiple Sclerosis (MS)		M1  polarization		 CD4^+^  CD8^+^		[337,338,339,340,341]
Amyotrophic Lateral Sclerosis (ALS)		M1  polarization	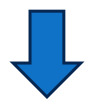	CONT for CD4^+^ CONT for CD8^+^  NK 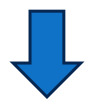 Treg		[257,342,343,344,345,346,347,348]
Huntington’s Disease (HD)		M1  polarization		 Th17.1  CD8^+^		[263,349,350,351,352]

## Data Availability

No new data were created or analyzed in this study. Data sharing is not applicable to this article.

## Abbreviations

5-HT	serotonin (5-hydroxytryptamine)
Aβ	amyloid-beta
AD	Alzheimer’s disease
AJs	adherens junctions
ALS	amyotrophic lateral sclerosis disease
ALT	alanine aminotransferase
AMPs	antimicrobial peptides
Ang-1, Ang-2	angiopoietins: -1 and -2
AOPP	advanced oxidation protein products
APC	antigen-presenting cells
AQP4	aquaporin 4
ARA	arachidonic acid
AST	aspartate aminotransferase
BBB	blood–brain barrier
BM	basement membrane
BMECs	brain microvascular endothelial cells
BMI	body mass index
BSHs	bile salt hydrolases
CLGI	chronic low-grade inflammation
CNS	central nervous system
COVID-19	coronavirus disease 2019
CRP	C-reactive protein
CSF	cerebrospinal fluid
CSFs	colony-stimulating factors
CVD	cardiovascular disease
CVOs	circumventricular organs
Cx43	protein connexin-43
DCs	dendritic cells
DD	death domain
DHA	docosahexaenoic acid
DII	dietary inflammatory index
DR4, DR5	death receptor-4 and -5
ecGCx	endothelial glycocalyx
ECM	extracellular matrix
ECs	endothelial cells
E-DII	energy-adjusted dietary inflammatory index
EGF	epidermal growth factor
EGFR	epidermal growth factor receptor
ENS	enteric nervous system
EPA	eicosapentaenoic acid
ER	endoplasmic reticulum
ERK	extracellular signal-regulated kinase
ESR	erythrocyte sedimentation rate
FFQs	food frequency questionnaires
GABA	gamma-aminobutyric acid
GBCAs	gadolinium-based contrast agents
GJs	gap junctions
GLUT1	glucose transporter type 1
GlycA	glycoprotein acetylation
Gp130	glycoprotein 130
H2O2	hydrogen peroxide
HBD-1, HBD-3	human β-defensins 1 and 3
HD	Huntington’s disease
HD5, HD6	human α-defensins 5 and 6
HDL	high-density lipoproteins
HOCl	hypochlorous acid
HPA axis	hypothalamus–pituitary–adrenal axis
hs-CRP	high-sensitivity C-reactive protein
HSDs	hydroxysteroid dehydrogenases
HSPG	heparan sulfate proteoglycan
IBD	inflammatory bowel disease
IES	inflammatory effect scores
IFN-γ	interferon-gamma
IIS	innate immune system
IL-1, IL-1β, IL-4, IL-6, IL-10, IL-12, IL-13, IL-22	interleukins: -1, -1β, -4, -6, -10, -12, -13 and -22
IL-1ra	interleukin-1 receptor antagonist
IL-6R	interleukin-6 receptor
JAK	Janus kinase
JAK1/2	Janus kinases 1 and 2
JAMs	junctional adhesion molecules
LAT1	L-type amino acid transporter 1
LDL	low-density lipoproteins
LPS	lipopolysaccharide
LTs	leukotrienes
M1 macrophage	proinflammatory polarized macrophage
Mφ	macrophage
MAPK	mitogen-activated protein kinase
MASLD	metabolic dysfunction-associated steatotic liver disease
mCRP	monomeric C-reactive protein (CRP)
MCs	mast cells
MDA	malondialdehyde
MHC	major histocompatibility complex
MIND diet	Mediterranean-DASH Intervention for Neurodegenerative Delay diet
MJL	Master Journal List
MLCK	myosin light chain kinase
MMPs	matrix metalloproteinases
MPO	myeloperoxidase
MRI	magnetic resonance imaging
mTNF-α	membrane-bound tumor necrosis factor-alpha (TNF-α)
MUC2	mucin 2
NDs	neurodegenerative diseases
NF-κB	nuclear factor kappa-light-chain-enhancer of activated B cells
NHANES	National Health and Nutrition Examination Survey
NK cells	natural killer cells
NLR	neutrophil-to-lymphocyte ratio
NLRP3	NOD-, LRR- and pyrin domain-containing protein 3 (NLRP3 inflammasome precursor)
NMR	nuclear magnetic resonance
NVC	neurovascular coupling
PAI-1	plasminogen activator inhibitor-1
PD	Parkinson’s disease
PDGFRβ	platelet-derived growth factor receptor-β
PET	positron emission tomography
PGs	prostaglandins
PKC	protein kinase C
PLR	platelet-to-lymphocyte ratio
PUFAs	polyunsaturated fatty acids
PV	plasma viscosity
Q-Alb	albumin quotient
Q-IgG	immunoglobulin G quotient
RA	rheumatoid arthritis
RME	receptor-mediated endocytosis
ROMs	reactive oxygen metabolites
ROS	reactive oxygen species
SAD	Standard American Diet
SCFAs	short-chain fatty acids
sIgA	secretory IgA antibody
sIL-6R	soluble interleukin-6 receptor (IL-6R)
SIRS	systemic inflammatory response syndrome
SIT	saturated influx transport
SLE	systemic lupus erythematosus
SNS	sympathetic nervous system
sPLA2	secretory phospholipase A2
SPMs	specialized pro-resolving mediators
STAT3	signal transducer and activator of transcription 3
sTNF-α	soluble tumor necrosis factor-alpha (TNF-α)
TAC	total antioxidant capacity
TACE	tumor necrosis factor-alpha converting enzyme, also known as ADAM17 (a disintegrin and metalloproteinase 17)
TEER	transepithelial electrical resistance
TGF-β	transforming growth factor-β
TJs	tight junctions
TLR2, TLR4	toll-like receptor 2 and 4, respectively
TLRs	toll-like receptors
TNF-α	tumor necrosis factor-alpha
TNFR1, TNFR2	tumor necrosis factor receptor 1 and 2, respectively
TRAIL	tumor necrosis factor-related apoptosis-inducing ligand, also known as
TNFSF10 or Apo2L	
UC	ulcerative colitis
UPR	unfolded protein response
VE-cadherin	vascular endothelial cadherin, also known as CD144 or cadherin-5
VEGF	vascular endothelial growth factor
WBC count	white blood cell count
WoS	Web of Science
YKL-40	glycoprotein YKL-40 [also known as chitinase-3-like protein 1 (CHI3L1) or human cartilage glycoprotein 39 (HC gp-39)]
ZO-1, ZO-2, ZO-3	zonula occludens: -1, -2, -3 (tight junction proteins)
